# Electrochemical Biosensors for the Detection of Antibiotics in Milk: Recent Trends and Future Perspectives

**DOI:** 10.3390/bios13090867

**Published:** 2023-09-01

**Authors:** Baljit Singh, Abhijnan Bhat, Lesa Dutta, Kumari Riya Pati, Yaroslav Korpan, Isha Dahiya

**Affiliations:** 1 MiCRA Biodiagnostics Technology Gateway, Technological University Dublin (TU Dublin), D24 FKT9 Dublin, Ireland; 2 Centre of Applied Science for Health, Technological University Dublin (TU Dublin), D24 FKT9 Dublin, Ireland; 3 Department of Chemistry, Central University of Punjab, VPO Ghudda, Bathinda 151401, Punjab, India; 4 Institute of Evolutionary Biology, School of Biological Sciences, University of Edinburgh, Edinburgh EH9 3JT, UK; 5 Institute of Molecular Biology and Genetics NAS of Ukraine, Department of Biomolecular Electronics, 03143 Kyiv, Ukraine; 6 Centre for Biotechnology, Maharshi Dayanand University (MDU), Rohtak 124001, Haryana, India

**Keywords:** antibiotics, biosensors, AMR, electrochemical instrumentation, nanomaterials, milk, immunosensors, MIPs, aptamers and enzymes

## Abstract

Antibiotics have emerged as ground-breaking medications for the treatment of infectious diseases, but due to the excessive use of antibiotics, some drugs have developed resistance to microorganisms. Because of their structural complexity, most antibiotics are excreted unchanged, polluting the water, soil, and natural resources. Additionally, food items are being polluted through the widespread use of antibiotics in animal feed. The normal concentrations of antibiotics in environmental samples typically vary from ng to g/L. Antibiotic residues in excess of these values can pose major risks the development of illnesses and infections/diseases. According to estimates, 300 million people will die prematurely in the next three decades (by 2050), and the WHO has proclaimed “antibiotic resistance” to be a severe economic and sociological hazard to public health. Several antibiotics have been recognised as possible environmental pollutants (EMA) and their detection in various matrices such as food, milk, and environmental samples is being investigated. Currently, chromatographic techniques coupled with different detectors (e.g., HPLC, LC-MS) are typically used for antibiotic analysis. Other screening methods include optical methods, ELISA, electrophoresis, biosensors, etc. To minimise the problems associated with antibiotics (i.e., the development of AMR) and the currently available analytical methods, electrochemical platforms have been investigated, and can provide a cost-effective, rapid and portable alternative. Despite the significant progress in this field, further developments are necessary to advance electrochemical sensors, e.g., through the use of multi-functional nanomaterials and advanced (bio)materials to ensure efficient detection, sensitivity, portability, and reliability. This review summarises the use of electrochemical biosensors for the detection of antibiotics in milk/milk products and presents a brief introduction to antibiotics and AMR followed by developments in the field of electrochemical biosensors based on (i) immunosensor, (ii) aptamer (iii) MIP, (iv) enzyme, (v) whole-cell and (vi) direct electrochemical approaches. The role of nanomaterials and sensor fabrication is discussed wherever necessary. Finally, the review discusses the challenges encountered and future perspectives. This review can serve as an insightful source of information, enhancing the awareness of the role of electrochemical biosensors in providing information for the preservation of the health of the public, of animals, and of our environment, globally.

## 1. Introduction

Antibiotics are a class of antimicrobial chemicals that are commonly used in medicine (both human and veterinary) for treating several infectious diseases [1,2]. The use of antibiotics is not limited to clinical conditions, but is also used in animal husbandry to treat animal diseases, to stimulate growth, to increase the efficiency of feed conversion and prevent diseases [3,4,5]. Veterinary antibiotics were first intended to cure and prevent infections, but they were subsequently introduced to animal feed to control the reproductive cycle, to increase breeding performance, as well as prophylactically and as growth promoters, considerably outweighing their usage as animal therapies [2,6]. Antibiotic selection and consumption patterns vary across continents and are influenced by food animal species, intensive or extensive farming, farming purposes, regional production patterns and types of production systems, a lack of a clear legislative frameworks or policies on antibiotic use, and the region’s size and socioeconomic status [7,8]. The use of unnecessary antibiotics in animal feed to promote growth is still completely unregulated in many developing and less-developed countries [9]. It is quite alarming that the antibiotics used in agriculture and veterinary care have similar or comparable goals, types, and modes of action to those recommended for use in people. In other words, they may be from same basic class(es), with the same function, and may behave in a similar fashion [10]. On a global scale, the average quantity of antimicrobial agents used yearly per kilogram of animal output varies by animal type; for cattle, this is 45 mg/kg [7]. Their method of administration also varies depending on the animal species. The expansion and increased concentration of farm lands, the ineffectiveness of government regulation and control with respect to the use and sale of antibiotics, decreased use of infection control techniques, and farmers’ resistance to implementing stipulated changes in farming practices are all factors contributing to the continued use of non-essential antibiotics in livestock farming [11]. To protect the health of the animals, boost output, and increase farmer income, antimicrobial agents continue to be used for growth promotion [12]. In general, farmers in developing nations buy antibiotics over the counter and use multidrug practices, which may have a significant influence on the volume and rate of their use in farming. Some of the primary factors contributing to this scenario include the high frequency of disease, the lack of state management policies, zone planning, and the insufficient application of hygiene practices in animal husbandry, as well as the utilisation of an integrated agricultural system [10,13]. Some of the antibiotics used in agriculture and animal husbandry (i.e., in cattle production) include oxytetracycline, streptomycin, penicillin, oxolinic acid, gentamycin, penicillin, tetracycline, ceftiofur, enrofloxacin, and tulathromycin, florfenicol, phenicol, lincosamide, tilmicosin, pleuromutilin, polypeptide, macrolide, carbadox, streptogramin, bambermycin, etc. [14]. The use of an integrated agriculture–aquaculture agricultural system, in which the aquaculture is supported by waste from both humans and cattle, increases the danger of exposing people, animals, and the environment to antibiotics [15]. Additionally, farmers rely on the guidance of local medicine vendors with respect to medication administration. Wealthy farmers, on the other hand, tended to use several antibiotics, as they can afford them [10,16,17,18,19].

Excessive antibiotic usage in veterinary and human medicine is a global issue that reduces the efficiency of antibiotics and leads to the development of antimicrobial resistance (AMR) [20]. Aside from treating infections caused by bacteria in food-producing animals, antimicrobials are frequently utilised in farming prophylactically and for growth promotion [21]. This is recognised as being unnecessary overuse in the EU (European Union), but although growth-promotion antibiotics have been banned, the prophylactic use of antibiotics still takes place and is still legal [22]. The emergence of AMR as a result of the overuse of antimicrobials has prompted serious global concern in recent years. The use of antibiotics in agriculture has been described as a major contributor to the clinical problem of disease resistance in human medicine [23]. Humans and animals can acquire resistant illnesses and commensal organisms simply by consuming them, which may then be transferred to the environment through the food chain [24,25]. There are no territorial or geographical boundaries to stop the development of antibiotic resistance, as the challenges are of local, regional, national, and international dimensions [26]. Over the next three decades, it is anticipated that 300 million people will die prematurely [27]. The World Health Organization has designated “antibiotic resistance” a severe social and economic danger to public health (WHO Guidelines) [28,29,30]. A number of antibiotics, e.g., tetracyclines, penicillin, fluoroquinolones, β-lactams, macrolides, aminoglycosides, amphenicols, lincosamides, glycopeptides and sulphonamides, have been identified as potential environmental contaminants (EMA Guidelines, 2015) and have been considered for detection in various matrices, such as in food, environmental and milk samples [31]. The WHO has designated numerous antimicrobials as “Critically Important Antibiotics” (CIAs) for humans that are also used to treat animal diseases. Due to the development of AMR, the abuse of CIAs may cause them to lose some of their effectiveness, making previously curable human illnesses potentially lethal [30]. The lack of adequate multi-sectoral and cross-disciplinary efforts among present means of combating AMR calls for swift and coordinated action in the context of the “One Health approach” [32]. The One Health concept includes initiatives to stop the improper use of antibiotics in people, food animals, and the environment. Approximately 70% of the antimicrobials supplied in the US are intended for use in food animals [33]. These medications are typically used to treat human diseases. A similar pattern can be seen in the data from 30 different European nations [34]. There is a lack of data from under-developed nations, but empirical estimates indicate that widespread use of antibiotics in food-producing animals is of serious concern [35,36].

In many countries, the dairy sector contributes significantly to overal agricultural production. Milk is used to make a variety of culinary items such as yoghurt, butter, cheese, cream, etc. The UK is the 13th-largest milk producer globally, and dairy/milk is the largest agricultural sector, contributing 16.4% of overall agricultural output (UK, 2020), worth GBP 4 billion [37]. Ireland produces more than 15% of the world’s infant milk formula, despite having less than 1% of the world’s dairy cows, with three of the world’s top producers operating here: Pfizer, Abbotts, and Danone. Agri-food exports have reached a new high (EUR 15.4 billion in 2021), accounting for 9.5% of overall Irish exports (Annual Review and Outlook for Agriculture, Food and the Marine 2022) [38]. The misuse of antibiotics has long-term consequences for human health and that of the environment. Antibiotics can infiltrate the water and land environment in various ways and remain as persistent organic pollutants as a result of antibiotic use and continuous emissions [2]. Antibiotic residues in foods (milk, meat, eggs, etc.) can cause a variety of toxic effects, including allergy, nephropathy, hepatotoxicity, mutagenicity, immunopathological effects, bone marrow toxicity, carcinogenicity, reproductive disorders, and anaphylactic shock [39,40]. However, the most common side effect of antibiotics in foods is the development of AMR, which has emerged as a severe global threat. Resistant bacterial diseases can be transmitted to humans via the food chain, rendering antibiotics and therapy ineffective [40,41]. To reduce the negative effects of veterinary drugs, including antibiotics, the EU has banned some specific antimicrobial growth promoters, and many regulatory authorities in the Europe and US have imposed Maximum Residue Limits (MRLs) [40]. In the EU, MRLs have been specified for all antimicrobial medicines permitted for veterinary use [21,42,43]. In the context of dairy farming, milk from antibiotic-treated cows is to be excluded from the supply chain in order to comply with legislation and the downstream industrial processes aimed at protecting human health. The minimum withdrawal periods for each drug between the last administration and the re-introduction of the milk to the supply chain have been defined [44]. Despite this, traces of antibiotics might be found in milk after the withdrawal period has passed [21]. Furthermore, several antibiotics have been proven to be stable across a wide temperature range, and even after pasteurisation (at temperatures of up to 100 °C) [45].

Screening technologies enabling sensitive and selective monitoring are necessary in order to guarantee that antibiotics/residues in animal-derived foods remain below the MRL. These methods can be classed as either conventional (such as immunological and microbiological methods) or modern. Although microbiological techniques are simple, but they have low sensitivity and specificity [46]. New technologies and recognition features underpin modern techniques [47]. To verify the results of screening methods, analytical techniques based on high-performance liquid chromatography (HPLC), liquid chromatography–mass spectroscopy (LC-MS), and gas chromatography–mass spectroscopy (GC-MS) have been used in parallel [48,49]. Analytical techniques are accurate, sensitive, and quantifiable, but require expensive equipment, expert personnel, and extensive sample pre-treatment and preparation times. To address the limits of traditional approaches, huge efforts have been made to create quick, sensitive, cost-effective, and portable biosensors and bioassays for on-the-spot antibiotics/residues detection in various matrices.

Biosensors monitor the chemical or biological processes as a function of the concentration of target analytes [50], and have been widely used in healthcare for monitoring blood glucose levels and the human chorionic gonadotropin (HCG) hormone in urine (i.e., pregnancy testing) [51]. Electrochemical approaches and nanomaterials including carbon-based metallic nanoparticles and composite materials, metal–organic frameworks, quantum dots, magnetic nanoparticles, etc., have been used to detect antibiotics/residues. To minimise the problems associated with antibiotics/AMR development and current analytical methods, electrochemical platforms that are cost-effective, portable and rapid could be an exciting alternative. However, despite the significant progress made in this field, further developments are necessary to advance electrochemical sensors, e.g., the use of multi-functional nanomaterials to ensure efficient detection, portability, and reliability. Electrochemical biosensors can be cost effective, rapid, easy to use and portable, which makes them attractive for on-site detection and monitoring of antibiotics.

To the best of our understanding, this review will provide a comprehensive summary of the use of electrochemical biosensors for the detection of antibiotics in milk, considering the latest developments and the relevant literature (mainly from 2010 onwards) in this area. This review presents an introduction to antibiotics and AMR followed by electrochemical biosensor approaches based on (i) immunosensors, (ii) aptamers, (iii) molecularly imprinted polymers (MIPs), and (iv) others (e.g., enzyme- and direct electrochemistry-based approaches). The role of nanomaterials or advanced functional biosensor fabrication materials is discussed wherever necessary. Finally, the paper analyses the issues faced by the currently available electrochemical biosensors, as well as the prospects for the future. We feel that this review will be a useful source of knowledge, increasing the understanding and appreciation of the importance of these electrochemical techniques in educating and safeguarding our animals and health worldwide.

## 2. Antibiotic Use and AMR Development

The principles and standards regarding the use of antibiotics in both clinical and agricultural contexts differ between developing and developed countries. Van Boeckel et al. [7] highlighted the fact that the patterns of variation in antibiotic use are influenced by the policies and regulations governing the manufacture, dispensation, and prescription of antibiotics [52]. Antimicrobial stewardship comprises the selection, dosage, route, and duration of antimicrobial agent administration: administration of the proper medicine at the correct dose, through the proper route, at the ideal time, to the appropriate patient, in order to achieve the optimal solution for prevention and treatment. To ensure the best results for treatment or prevention, antimicrobial stewardship involves selecting the appropriate antimicrobial agent to be used, as well as the right dosage, route, patient, and time of administration [53]. Optimising therapeutic outcomes, maximising clinical treatment and prevention, and minimising the unintended penalties and consequences of antibiotic usage (including toxicity and the emergence of antibiotic-resistant bacteria) are the main goals of antimicrobial stewardship [53,54].

At both the personal and the societal levels, there is a connection between antibiotic usage and resistance. Poor sanitation and hygiene conditions, disparities in healthcare systems, lax antibiotics policies (which affect the potency and quality of the drugs produced), over-the-counter drug purchases and unregulated prescription principles (encourage self-medication and prescription by untrained people), patient expectations, and financial incentives for healthcare providers are some of the factors that contribute to high disease burdens [52,55,56]. The public health system is currently under strain due to growing urbanisation, resulting in ecological health (sanitation and air quality) being compromised [57]. Scientific papers have increasingly highlighted how the environment has affected the rise of AMR, but surveillance systems and health policy frequently fall short of addressing this issue [58].

**Figure 1:**
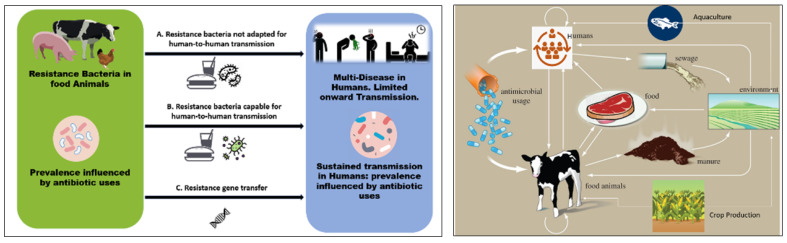
(**Left**) Schematic illustration of possible links between antibiotic use in agriculture and human disease. The usage of antibiotics has an impact on the incidence of resistant microorganisms. The capacity for sustained human-to-human transmission is a critical factor in the impact of infection. Arrows connecting the two populations show: (**A**) direct transmission of bacteria that are not adapted for transmission to humans via the food chain (e.g., campylobacter, salmonella); or (**B**) direct transmission of organisms that are adapted for transmission to humans; and (**C**) transfer of resistance genes from the agricultural setting into pathogens that are transmitted among humans. (**Right**) Pathway of antibiotics and antibiotic-resistant genes through agriculture and livestock.

Figure 1 presents a schematic illustration of the potential connections between the use of antibiotics in agriculture and in human disease (left) and the transfer of antibiotics and antibiotic resistance genes through agriculture and cattle (right). Once resistant bacteria associated with animals have been developed, they can enter the food chain through the consumption of meat and animal products. According to Woolhouse et al., there are four primary perspectives from which to examine antibiotic resistance in livestock/farming: the animals (cattle, sheep, etc.), products from animals, farm personnel, and environmental locations (water, wastewater, lagoon, sewage, soil, manure, feeds, and sludge after treatment) [59]. These make up various compartments and niches within the farm (ecosystem). Like humans, animals have a varied population of microorganisms living in their digestive tracts, including resistant bacteria and commensals, and this acts as the most significant reservoir of microorganisms, and may be extremely important for the spread and acquisition of resistant bacteria and their genes [60]. According to Van et al., eating food contaminated with antibiotic-resistant bacteria can amplify resistance genes, making it easier for the antibiotic resistance determinants to spread to other bacteria that are important to human health, and potentially even increasing the spread of pathogenic bacteria among humans [61,62].

Manure, which has been identified as a hotspot for antibiotic-resistant bacteria and genes, can act as a feasible pathway for antibiotic-resistant bacteria and genes to enter the soil and water through purposeful or unintentional processes [63]. Therefore, manure should be treated using biological methods, such as anaerobic digestion, before being applied to the soil. As a result, it is crucial to post-treat digestate using chemical, physical, mechanical, and biological techniques [64]. Prior to digestate being applied to land, the microbiological risk to safety and sanitation should also be assessed in an effort to avoid negative ecological, animal, environmental and human health effects. Taking actions to control the misuse of antibiotics in animal production will have a significant impact on antibiotic resistance levels due to the interactions among the environment, animals, and humans.

The following points would be useful to control the use of antibiotics in agriculture or agricultural animals [7,10,15,65]:

1. Develop and implement on-farm antibiotic stewardship programs: On-farm antibiotic stewardship programs could be developed and implemented to promote the appropriate use of antibiotics in food-producing animals. These programs could involve education and training for farmers and veterinarians, guidelines for antibiotic use, monitoring of and feedback regarding antibiotic use, and the use of alternative approaches to disease prevention, such as improved animal husbandry practices and vaccination.
2. Implement regulations to restrict use of antibiotics for growth promotion: Regulations could be put in place to prohibit the use of antibiotics for growth promotion and other non-therapeutic purposes in food-producing animals. This could help to reduce the overall use of antibiotics in animals and agriculture and prevent the development of AMR. Pharmacists and veterinary officers should adhere strictly to the regulations and policies governing prescriptions. Policymakers should enact strict controls and regulations to ensure that antibiotics used in farming are only purchased legally (legitimately). Antibiotic usage in animals should be decreased and limited by utilising plant-derived extracts and probiotics/prebiotics for disease prevention and treatment.
3. A reduction in the antibiotics required via improvements in animal health and welfare: Efforts could be made to improve animal health and welfare through better housing, nutrition, and disease prevention measures. This could help in reducing the need for the use of antibiotics in food-producing animals. By implementing such measures, it would be possible to control use of antibiotics in agricultural animals and reduce the development of antibiotic resistance. The government and financially stable companies should encourage and support the employment of regular, efficient and appropriate veterinarian services.

## 3. Detection Methods (Antibiotics)

**Figure 2:**
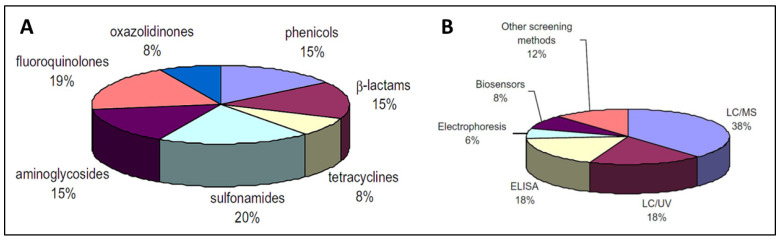
Distribution (**A**) and classification (**B**) of the analytical methods used for antibiotics determination in food [66]. Reprinted from Ref. [66], Copyright (2010), with permission from Elsevier.

Due to the increasing concern regarding AMR, various methods for determining antibiotic levels in food items have been developed, including colorimetry, fluorescence, chemiluminescence, surface-plasmon resonance (SPR), mass-sensitive and electrochemical biosensors. Antibiotic detection methods can be classified as screening (semi-quantitative) or confirmatory (quantitative). Confirmatory techniques depend heavily on LC-MS to identify antibiotics/residues. LC-UV or electrophoresis have also been reported [66]. Screening methods are semi-quantitative and are feasible due to the minimal possibility of false-positive data, as well as their speedy analysis, cost effectiveness, ease of operation, and selectivity. Figure 2 presents the distribution of analytical methods for the detection of antibiotics in food [66]. Biosensors seems to be a highly feasible approach for large-scale antibiotic residue detection.

## 4. Electrochemical Instrumentation and Nanomaterials

Due to their benefits such as superior sensitivity, speed, and miniaturisation capability through combinations of potential (E), current (I), charge (Q), and time (t), electrochemical sensors have recently shown significant promise in the detection of antibiotics/residues. Typically, in an electrochemical cell, three electrodes are used (although two-electrode or four-electrode systems can also be used): the working electrode, the reference electrode and the counter electrode. The working electrode (carbon, gold, or platinum) is generally modified using biorecognition elements to achieve sensitive and selective transduction of the analyte of interest. The counter and reference electrodes control the potential of the working electrode and provide a path for the current produced. Measurement is performed using a potentiostat. The working electrode is a core component of electrochemical sensors, the behaviour of which determines the sensor performance.

**Figure 3:**
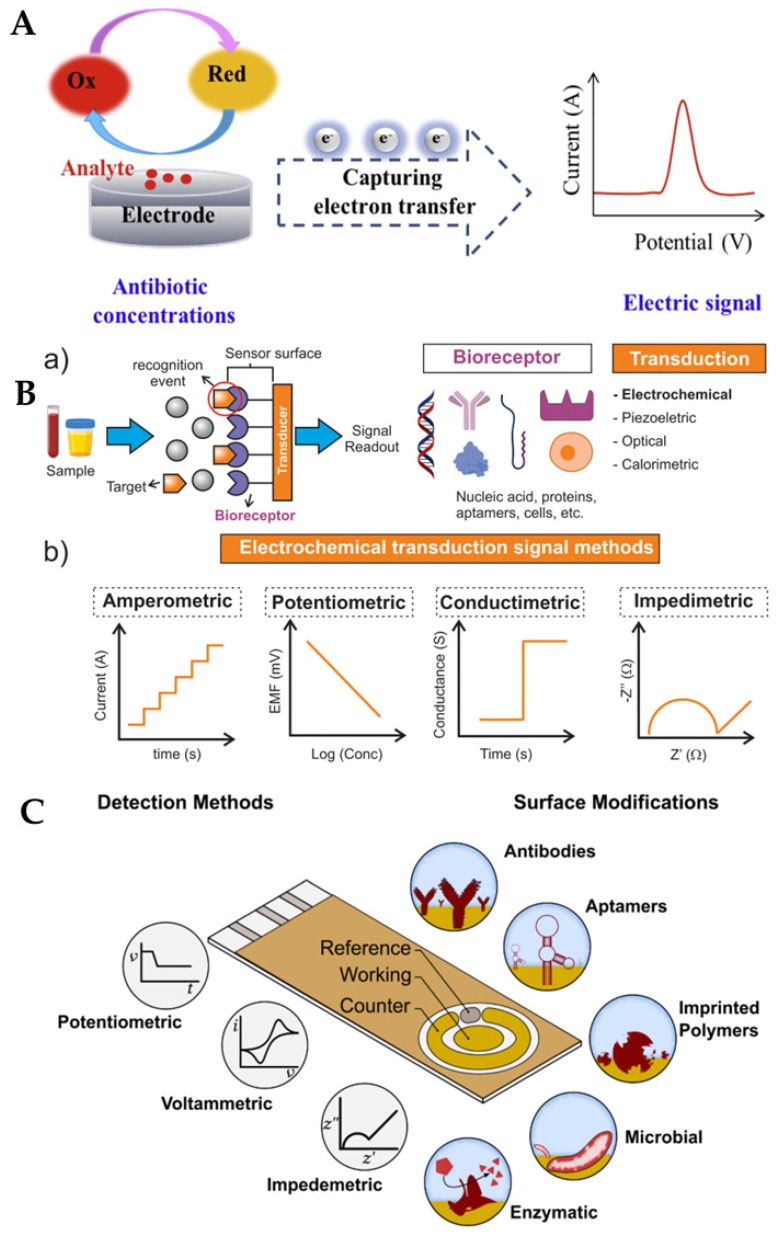
(**A**) Principle of electrochemical sensors (electrode) for the detection of antibiotics [68]. Reprinted from Ref. [68], Copyright (2021), with permission from Elsevier. (**B**(**a**)) Schematic of a generic biosensor device. A sample containing the target (analyte) is placed in close contact with the surface of the sensor. The bioreceptor (antibody, lectin, enzyme, aptamer, DNA, or cells), which is immobilised above the transducer (electrochemical, piezoelectric, optical, calorimetric modes, etc.), binds with the target from the sample (recognition event), after which the transducer converts the binding event into a measurable signal that is proportional to the concentration of the target (signal readout). (**b**) Examples of electrochemical transduction signals encompass amperometric, potentiometric, conductimetric, and impedimetric signals [69]. Reprinted from Ref. [69], Copyright (2022), with permission from Elsevier. (**C**) Electrochemical detection, in which the working, reference and counter electrodes are arranged in a manner allowing the sample to be in contact with all three at the same time. The range of possible surface modifications on the working electrode are shown [70]. Reproduced under the terms of the CC-BY licence from Ref. [70], Copyright 2021, The Authors, published by MDPI.

The major electroanalytical methods include potentiometry, voltammetry (and polarography), amperometry, coulometry, conductometry and electrogravimetry. The names of these electroanalytical methods reflect either the electrical/electrochemical property being measured or the units employed. Typically, voltammetric methods rely on the measurement of the resulting current of the oxidation/reduction process at the electrode surface. The simplest form of voltammetry involves the application of a constant potential and measuring the resulting current over a specific time (amperometry, current vs. time) and integrating it over a period of time (coulometry, charge vs. time). In cyclic voltammetry (CV), the electrode potential ramps linearly versus time in cycles or cyclical phases. Other advanced electroanalytical techniques, such as differential pulse voltammetry (DPV) and square-wave voltammetry (SWV), can be applied by by varying potential over time in order to improve the signal-to-noise ratio or for the analytical measurement of the redox reaction. Square wave voltammetry, differential pulse voltammetry, cyclic voltammetry, and stripping voltammetry are used extensively for the electrochemical detection of antibiotic residues. Other electroanalytical techniques, such as electrochemical impedance spectroscopy (EIS) and chronoamperometry (CA), have also gained popularity for antibiotic detection. The principles of electrochemical sensors, a schematic for electrochemical biosensor detection methods, and examples of electrochemical transduction signals (potentiometric, amperometric, conductimetric and impedimetric signals) are presented in Figure 3 [67,68,69,70]. We recently reported detailed information on a variety of electroanalytical techniques in another publication [71].

**Figure 4:**
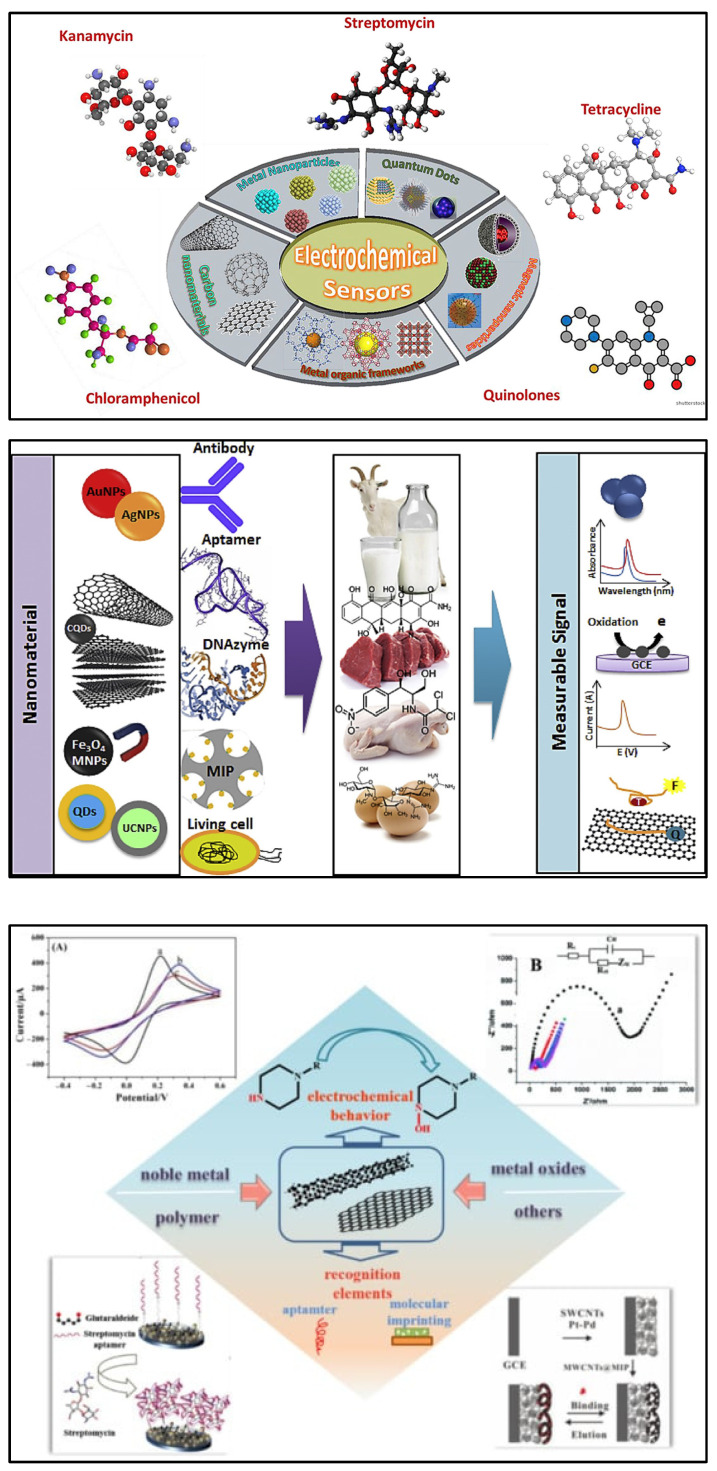
(**Top**) Recent advances in nanomaterial-based electrochemical detection of antibiotics (graphical representation) [72]. Reprinted from Ref. [72], Copyright (2020), with permission from Elsevier. (**Middle**) Schematic illustration for application of various nanomaterials and biorecognition elements in the development of antibiotic biosensors [73]. Reprinted from Ref. [73], Copyright (2020), with permission from Elsevier. (**Bottom**) An overview of functional carbon nanomaterials and their application in the detection of antibiotics [74]. Reproduced under the terms of the CC-BY licence from Ref. [74] [*Nanomaterials*], Copyright 2022, The Authors, published by MDPI.

Nanomaterials are considered a promising option in the design and fabrication of so-called “functional” transducers for electrochemical biosensing systems due to unique physical and chemical characteristics. Different nanostructures including carbon-based nanomaterials, metallic nanoparticles, quantum dots (QDs), magnetic nanoparticles (MNPs), etc., are integrated into the biosensing for antibiotics. The flexibility of such systems due to their potential for functionalisation and post-modification has helped to dramatically improve their analytical performance in the detection of antibiotics. Recently, significant attention has been given to various nanomaterials employed for the electrochemical sensing of antibiotics (Figure 4) [72,73,74,75].

## 5. Electrochemical Biosensors

**Figure 5:**
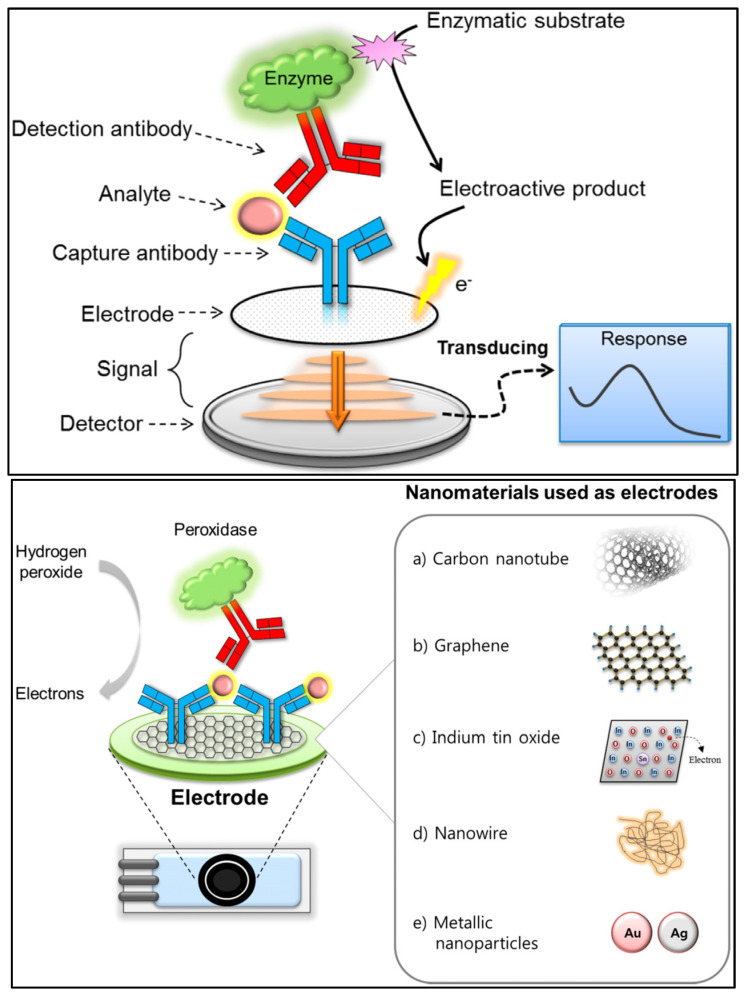
(**Top**) Basic analytical principle of electrochemical immunosensors. (**Bottom**) Nanomaterials used as electrodes or supporting solid matrices to enhance the analytical performance of electrochemical immunosensing [84]. Reproduced under the terms of the CC-BY licence from Ref. [84], Copyright 2018, The Authors, published by MDPI.

Electrochemical biosensors are often used due to their affordable cost, fast analysis time, portability, and potential for miniaturisation [76,77,78,79,80], as well as their well-reported suitability for the detection of antibiotics [42,70,74,81,82,83]. Various strategies can be used to transduce (electrochemically) a biorecognition reaction with antibiotics (shown in Figure 5). This review covers the research and development of electrochemical biosensors for antibiotic detection in milk, and approaches based on (i) immunosensors, (ii) aptamers, (iii) molecularly imprinted polymers (MIPs), (iv) enzymes, (v) whole cells, and (vi) direct electrochemistry are discussed below.

### 5.1. Electrochemical Immunosensors

In electrochemical immunosensors, an antibody is used as a capturing agent, and the electrical signal produced by the binding of the antibody to the target analyte is measured [84]. The electrochemical immunosensing principles can be classified as either amperometry, potentiometry, impedance or conductometry, depending on the specific signals being measured. Better loading capacity and mass-transport abilities are produced by nanomaterials with larger surface areas, which synergistically contribute to signal amplification. Electrochemical immunosensors provide excellent reliability (due to specific antigen–antibody recognition), sensitivity and cost effectiveness [85,86]. However, it is necessary for electron mediators to be integrated in order to be able to electrochemically utilise the extremely specific antigen–antibody interactions [87]. Because electrochemical immunosensors typically lack the requisite sensitivity to detect very low levels of antibiotic residues, major efforts have been made towards the development of novel nanomaterials to achieve effective biomolecule immobilisation on the electrode surface. Various nanomaterials have been reported to carry out the immunosensing of label-free antibiotics (thus improving sensitivity), including carbon nanomaterials (carbon nanotubes and graphene) [88,89], nanoporous gold [87], polymers (poly pyrrole-N-Hydroxysuccinimide) [90], magnetic nanomaterials [91], quantum dots [92] and dye molecules [93,94].

**Figure 6:**
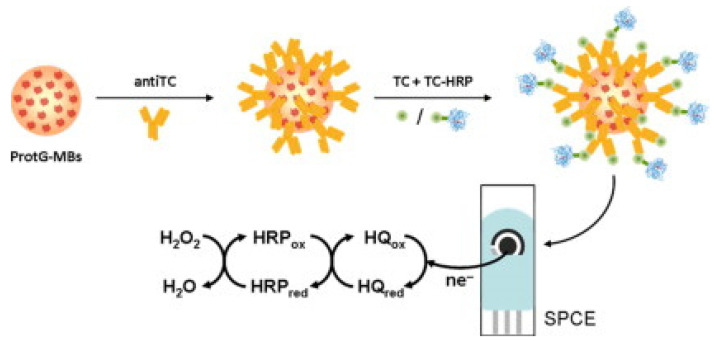
Graphical representation/abstract of the disposable amperometric magneto-immunosensor for the direct detection of tetracycline antibiotic residues in milk [95]. Reprinted from Ref. [95], Copyright (2012), with permission from Elsevier.

An immunosensor for the determination of tetracycline was proposed by Conzuelo et al.: an amperometric magneto-immunosensor was fabricated by immobilising selective antibodies on a carbon SPE surface modified with magnetic beads functionalised with protein G [95]. The immunoassay was based on the competitive binding between the HRP-labelled tracer and the antibiotic to an antibody. Hydrohen peroxide (H_2_O_2_) in the presence of hydroquinone was used as a substrate, followed by amperometric measurements (Figure 6). The performance of the sensor was analytically evaluated towards oxytetracycline, chlortetracycline, tetracycline, and doxycycline. The dynamic range and LOD for tetracycline were found to be 17.8–189.6 ng/mL and 8.9 ng/mL, respectively. Selectivity was evaluated against six non-target antibiotics commonly present in milk/dairy products, and no significant cross-reactivity was observed. The sensor’s usefulness was tested by analysing diluted (1:1) whole milk solution spiked with tetracycline (mean recovery of 99%). The authors claimed that the developed magneto-immunosensor enabled sensitive and specific tetracycline detection in milk [95]. El-Moghazy et.al reported a label-free immunosensor for the amperometric detection of chloramphenicol residues (in milk) using the carbon electrode. The immunosensor exhibited good sensitivity, with an LOD 0.0047 ng/mL and a range of 0.01–10 ng/mL [96].

Kling et al. reported a multi-target microfluidics-based platform for simultaneous electrochemical detection of tetracycline and streptogramin using up to eight enzyme-linked assays (ELAs) [83]. Microfluidic channel networks comprising various (distinct) immobilisation sections (680 nL each), each of which was passively metered, could be separately actuated for an individual assay. The determined LODs for the simultaneous dection of tetracycline and pristinamycin were 6.33 ng/mL and 9.22 ng/mL, respectively, in human plasma (spiked), within a timeframe of 15 min. The channel (dry film photoresist, DFR) material was reported to provide easy storage for pre-immobilised assays, with a shelf-life of 3 months [83].

**Figure 7:**
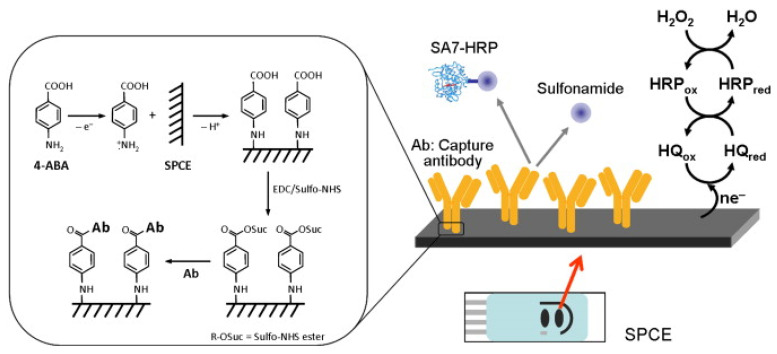
Schematic display of the immunosensor developed for the detection of sulfonamide antibiotics (inset: details of the surface chemistry involved in the covalent immobilisation of the capture antibody by using EDC and Sulfo-NHS 4-ABA film formed on an SPCE) [97]. Reprinted from Ref. [97], Copyright (2012), with permission from Elsevier.

Felipe Conzuelo and his team developed a screen-printed carbon electrode-based integrated amperometric immunosensor for sulfonamide detection in milk [97]. The amperometric response to the addition of hydrogen peroxide (H_2_O_2_) in the presence of hydroquinone (HQ) was evaluated at −0.2V against the silver pseudo-reference electrode. They claimed the ability to detect low-level sulfonamide residues in milk samples (diluted with assay buffer). An LOD <1 ng/mL was achieved, which is lower (by two orders of magnitude) than the maximum level allowed for total sulfonamides (Figure 7) [97].

**Figure 8:**
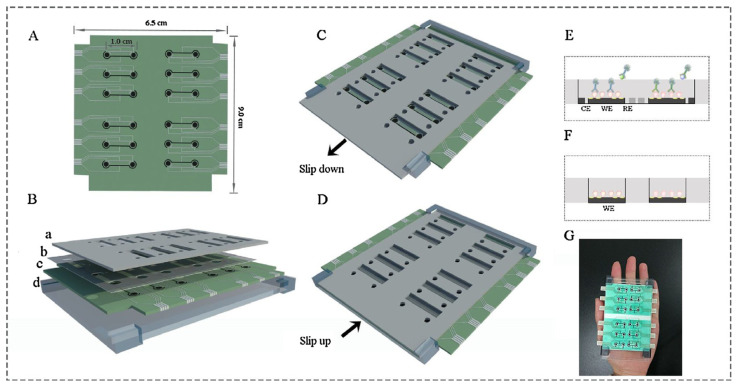
Layout of 24-site fluidic micro-array analytical device: (**A**) one-dimensional schematic plot of the base layer; (**B**) three-dimensional layered schematic plot of the whole device; (**C**) detection state of the Isensor; (**D**) preparation state of the Isensor; (**E**) micro-reservoir of one analysis unit in its detection state; (**F**) micro-reservoir of one analysis unit in its preparation state; (**G**) picture of the analytical device [98]. Reprinted from Ref. [98], Copyright (2018), with permission from Elsevier.

**Figure 9:**
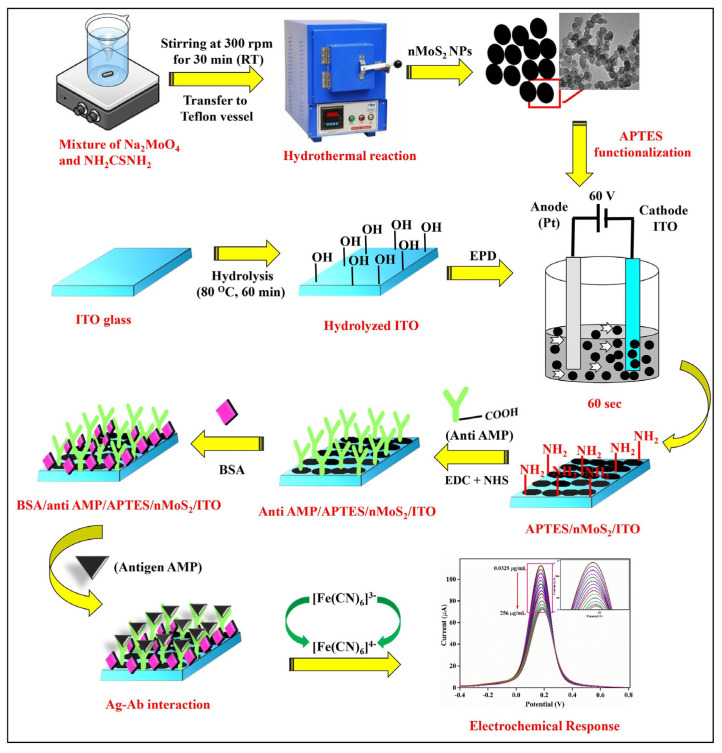
Schematic representation of the synthesis of nMoS_2_ NPs and the development of BSA/anti-AMP/APTES/nMoS_2_/ITO immunoelectrode for AMP detection [99]. Reprinted from Ref. [99], Copyright (2021), with permission from Elsevier.

Ying Gu and his group proposed a multiplex and co-instantaneous immunosensing approach by constructing an unique 24-site fluidic microarray screen-printed electrochemical device based on a double-deck slide construction and an assembled micro-reservoir (Figure 8) [98]. Bimetallic Au@Pt mesoporous nanoparticles were applied for effective analysis of enrofloxacin and melamine with linear ranges of 0.1–1000 ng/mL and 0.1–500 ng/mL and LODs 18.97 pg/mL and 26.80 pg/mL, respectively [98]. Amit K. Yadav and their group introduced an electrochemical immunosensor for ampicillin detection using conductive amine-functionalised nanostructured MoS_2_-based nanoparticles as an immobilisation platform (Figure 9) [99]. The authors claimed it to be a convenient and label-free-sensitive system for the analysis of food samples, and the fabricated immunosensor showed a low LOD of 0.028 μg/mL and a linear detection range of 0.0325–64 μg/mL. Table 1 summarises some further examples of electrochemical immunosensors for the detection of antibiotics (in milk).

**Figure 10:**
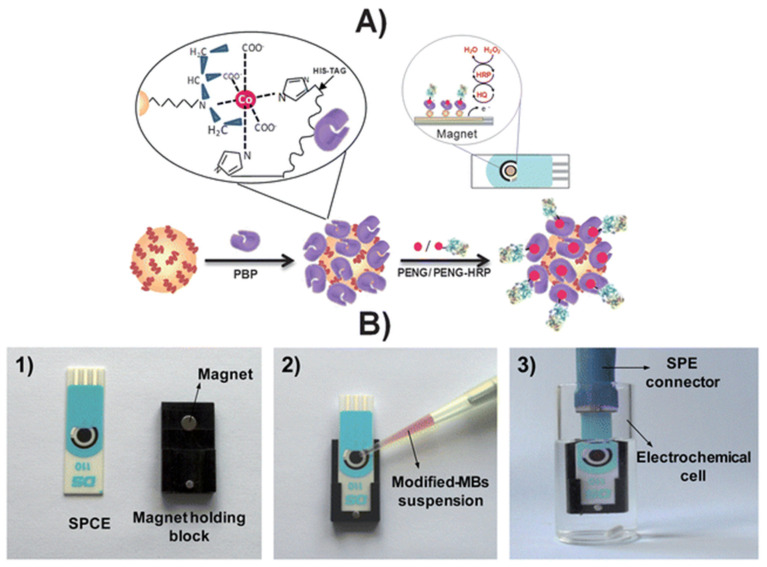
(**A**) Schematic display of the steps involved in the affinity magnetosensor developed for β-lactam antibiotics. (**B**) Picture showing the SPCE and the home-made magnet holding block (**1**), the deposition of the modified MBs on the SPCE assembled on the magnet holding block (**2**) and the assembled SPCE-magnet holding block immersed in the electrochemical cell used for the amperometric measurements (**3**) [100]. Reproduced from Ref. [100], Copyright (2013), with permission from the Royal Society of Chemistry.

An affinity magnetosensor (amperometric, based on screen-printed carbon electrodes (SPCEs) and recombinant penicillin-binding protein (PBP)) for the detection of β-lactam residues (in milk) has been reported. PBP was immobilised using magnetic beads modified with His-Tag-Isolation, and a competitive assay using a tracer with HRP (enzymatic label) was performed. The response of SPCE at −0.20 V (vs. silver pseudo-reference) upon H_2_O_2_ addition (in the presence of HQ) was used as a signal. The method showed low ppb level limits of detection for the six antibiotics tested (milk samples, untreated)—penicillin G sodium salt (PENG), amoxicillin (AMOX), ampicillin sodium salt (AMPI), cefapirin (CEF), oxacillin (OXA), and cloxacillin (CLOX)—and reported reasonable (good) selectivity against other antibiotics/residues frequently detected in dairy and milk products. The proposed methodology detected the active form of β-lactams (high affinities for penicillins and cephalosporins, likely because of the bioreceptor used), with an analysis time of 30 min (Figure 10) [100].

**Table 1:** Electrochemical immunosensors for the detection of antibiotics (in milk).

Antibiotics	Bio Recognition Component	Working Electrode	Detection Method/Technique	Linear/Dynamic Range (ppb)	LOD (ppb)	Label	Sample Type	Ref.
Sulfapyridine	Polyclonal antiserum As167	Ab covalently immobilized on 4-ABA-modified SPCEs	Amperometry	1.6 to 118.6	0.44	HRP	Dilutedwhole milk	[97]
Sulfamethoxazole	anti-sulfamethoxazole polyclonal antibody	antiSMX/nanoCeO_2_—CS/GCE	DPV	0.5 to 500	0.325	HRP	Buffer/ food samples	[101]
Tetracyclines	Polyclonal sheep antiTC antibody. Competitive immunoassay using TC-HRP on antiTC-modified MBs	ProtG-MBs /SPCE/H_2_O_2_ in the presence of HQ	Amperometry	5.0 to 202.5	1.9	HRP	Undiluted milk	[95]
Cefquinome	BlaR-CTD	GO/TH/GCE	CV & EIS	0.1 to 8	0.16	HRP-AMP	PBS/milk	[102]
Ampicillin	Electrochemical immunosensor	BSA/anti-AMP/APTES/nMoS_2_/ITOimmunoelectrode	DPV	32.5 to 64 × 10^3^	28	Label-free	PBS/spiked milk, orange juice & tap water	[99]
Ciprofloxacin	11-BSA-MB, 11-HRP and Ab171-MB	Amperometric magneto-immunosensor (AMIS)	Amperometry	0.043 to 7.38	0.009	HRP	Whole milk	[103]
Enrofloxacin	Electrochemical immunosensor	rGO-TEPA/SPE	DPV	0.1 to 1000	1.897 × 10^−2^	Dendritic mesoporous Au@Pt nano-probe	PBS/spiked milk samples	[98]
Chloramphenicol	Antibody/immunosensor	PVA-co-PE NFM/Anti-CAP/SPCE	Amperometry	0.01–10	0.0047	label-free	PBS/spiked milk	[96]
Penicillin G	Antibody/immunosensor	AP/gold/s-BLM/GC	EIS	3.34 × 10^−6^ to 3.34	2.7 × 10^−7^	gold/s-BLM	Diluted milk	[104]

### 5.2. Aptamer-Based Electrochemical Biosensors

Aptamers are either bonded by covalent bonds or adsorbed physically on the electrode surface, in addition to other supporting components (e.g., mercaptohexanol), in order to avoid analyte adsorption. In addition to conventional immobilisation techniques, aptamers can be deposited directly onto the sensor surface, and the hybridisation of aptamers with complementary immobilised DNA strands has also been reported [105]. The covalent binding of the aptamer occurs via the terminal functional groups, and is related to the underlying material. Through the creation of Au-S bonds, thiolated aptamers are immobilised on gold nanoparticles decorated onto the surface or on bare gold electrodes, while aminated aptamers attach covalently to carboxylated substrates (e.g., carbon nanomaterial-based supports) [106,107,108]. Recently, the electron grafting of the electrode with diazonium salt has become popular (formed from p-aminobenzoic acid or p-aminophenylacetic acid) [109], where the precursor is treated with nitrite and then electrodeposited. The carboxylic functional groups are used in the reaction with carbodiimide to immobilise the aminated aptamers (glutaraldehyde crosslinking) [110]. Glutaraldehyde binding is advantageous, e.g., for the application of aminated carriers (poly(amidoamine), PAMAM dendrimers), which help to increase the surface density of the aptamers.

**Figure 11:**
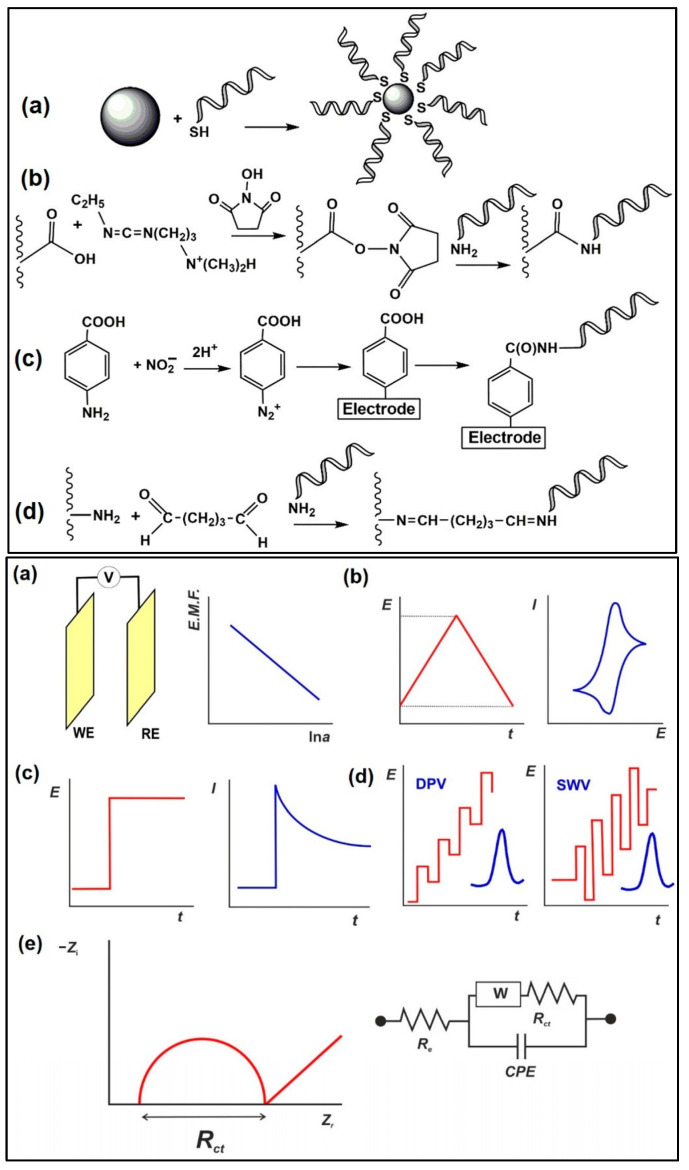
(**Top**) Immobilisation protocols applied in electrochemical aptasensors: (**a**) interaction of thiolated aptamer with AuNPs/bare Au electrode; (**b**) carbodiimide binding to carboxylated support; (**c**) electrografting with diazonium salt generated from aromatic amino group; (**d**) glutaraldehyde cross-binding of aminated aptamer and carrier. (**Bottom**) Electrochemical sensors used as transducers of aptasensors: (**a**) potentiometric cell: WE—working electrode, RE—reference electrode, V—voltmeter; (**b**) cyclic voltammetry; (**c**) amperometry; (**d**) differential pulse voltammetry (DPV) and square wave voltammetry (SWV). The red line illustrates the shape of the current peak obtained; (**e**) electrochemical impedance spectroscopy; the Nyquist diagram and equivalent circuit correspond to the double-electric layer. *R_e_* is electrolyte resistance, *R_ct_* is charge transfer resistance, *W* is Warburg impedance, and *CPE* is the constant phase element [111]. Reproduced under the terms of the CC-BY licence from Ref. [111] [*Sensors*], Copyright 2022, The Authors, published by MDPI.

Schemaitc diagrams of the immobilisation protocols are outlined in Figure 11 [111]. Compared to antibodies, aptamers are more physicochemically stable, and have a longer shelf-life, with reasonable cost, easy synthesis (in vitro), and relatively simple modification procedures [112,113]. There are two traditional approaches for immobilising aptasensors: (i) non-covalent modification of functionally activated surfaces, and (ii) direct modification of a bio-functionalised sensor surface using suitable linkers. Nanomaterials are often incorporated in electrochemical detection of antibiotics to improve the sensitivity of the aptasensors. The use of methylene blue (MB)-, ferrocene- and heavy-metal ions (Pb^2+^ and Cd^2+^)-doped metal–organic frameworks has been reported as aptasensors to achieve sensitive and distinctive multiplexed antibiotic detection [114,115,116].

**Figure 12:**
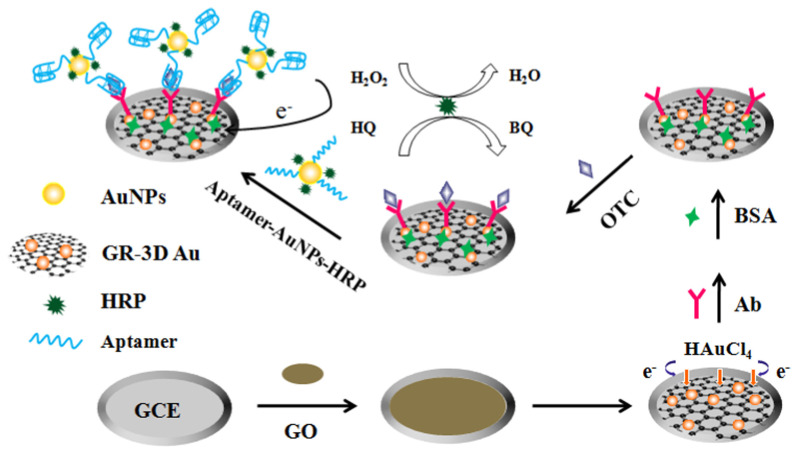
Schematic representation of the sandwich-type aptasensor based on GR-3D Au and aptamer-AuNPs-HRP for the detection of oxytetracycline [117]. Reprinted from Ref. [117], Copyright (2017), with permission from Elsevier.

Liu et al. proposed an electrochemical aptasensor based on sandwich assay for oxytetracycline detection (Figure 12) [117]. The aptasensor used a graphene three-dimensional nanostructured gold nanocomposite and aptamer-AuNPs-HRP nanoprobes for signal enhancement, and demonstrated reasonable performance and achieved an LOD of 4.98 × 10^−4^ ng/mL. Mohammad-Razdari and his group [118] developed an electrochemical aptamer-based biosensor that used a mix of magnetic nanoparticles (MNP) and gold nanoparticles (GNP) to detect tetracycline residues with a linear concentration range of 1.0 pM–1.0 M and a limit of detection of 0.03 pM. These values were lower than the value permissible in the European Union (225 nM). The aptasensor’s repeatability was determined to be 5.9% (RSD) [118].

**Figure 13:**
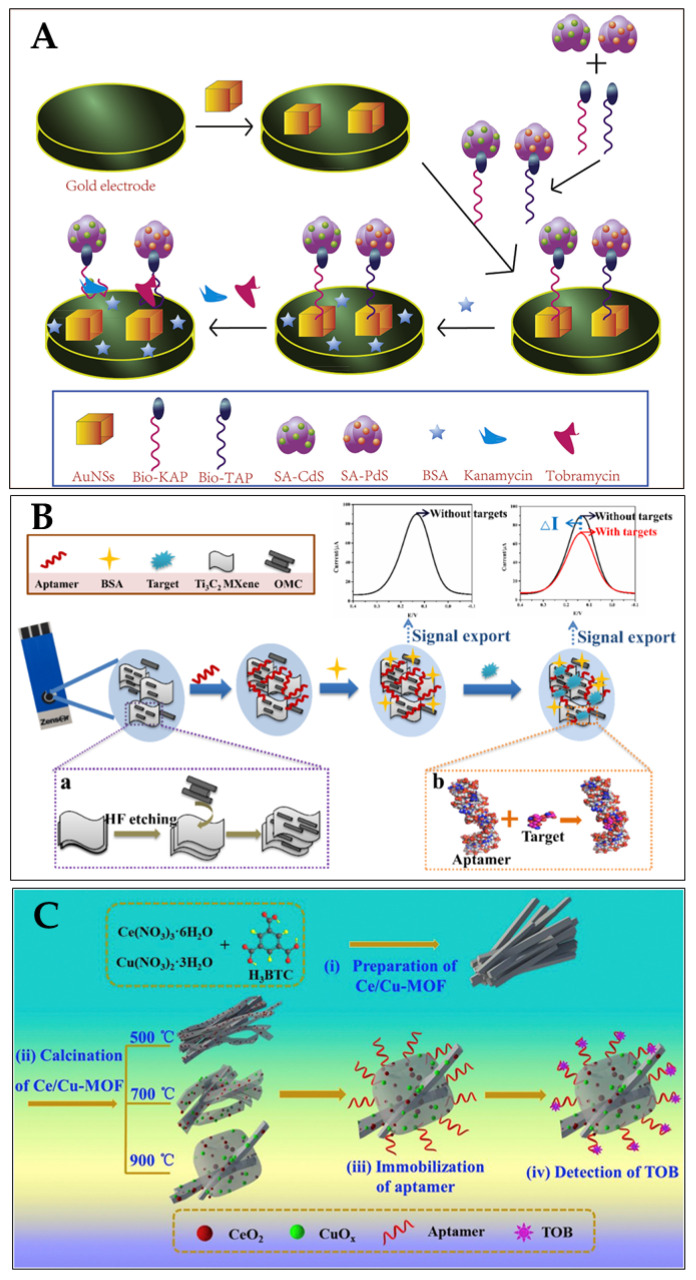
(**A**) Schematic illustration of aptasensor based on the utilisation of QDs, AuNSs, RNA-based aptamer strands, and high-affinity pairing between Bio and SA for the simultaneous detection of multiple antibiotics [119]. Reprinted from Ref. [119], Copyright (2021), with permission from Elsevier. (**B**) Schematic aptasensor fabrication process: (**a**) formation process of OMC@Ti_3_C_2_ MXene; (**b**) binding mode of aptamer with target [120]. Reproduced under the terms of the CC-BY licence from Ref. [120], Copyright 2022, The Authors, published by MDPI. (**C**) Schematic diagram of the fabrication procedure of the CeO_2_/CuO_x_@mC-based aptasensor for detecting TOB, including (**i**,**ii**) the preparation of the series of CeO_2_/CuO_x_@mC nanocomposites, (**iii**) the immobilisation of the aptamer strands over the CeO_2_/CuO_x_@mC composite, and (**iv**) TOB detection using the proposed CeO_2_/CuO_x_@mC-based aptasensor [121]. Reprinted from Ref. [121], Copyright (2019), with permission from Elsevier.

Tobramycin (TOB) and Kanamycin (KAN) were employed as model analytes to build a dual-labelled aptasensor for the simultaneous detection of antibiotics (Filan Li and his team, Figure 13A) [119]. Due to their exceptional surface-to-volume ratio, the AuNSs had a high loading efficiency for the two composites formed on a gold-electrode surface. The DPV signals were amplified, as many metal-ion labels were dissolved in the solution. The aptasensor showed a broad linear range (KAN: 1–400 nM and TOB: 1–10,000 nM) and low limits of detection (KAN: 0.12 nM and TOB: 0.49 nM) in spiked milk samples [119].

For the simultaneous detection of aminoglycoside antibiotics, an aptasensor based on an SPCE (screen-printed carbon electrode) was reported by Fengling Yue and his team (Figure 13B) [119]. They used an aptamer as a bio-recognition element (AAs). As a nanocarrier, the synthesised OMC@Ti_3_C_2_ MXene held a large number of aptamers. The bio-chemical reactions between the aptamer and AAs affected the transfer of electron charge on the SPCE surface, leading to a notable decrease in the DPV values. A limit of detection of 3.51 nM was achieved [120]. Shijun Wang and his team described (Figure 13C) a cerium/copper-base bimetallic metal–organic framework (Ce/Cu-MOF) and derivatives, and used this as a scaffold for an electrochemical aptsensor for tobramycin (trace) detection in milk and human serum. Upon high-temperature calcination, the Ce and Cu coordination centers were transferred to oxides with different chemical valences (Cu(II), Cu(0), Ce(III) Ce(IV)), embedded in a mesoporous carbon network derived from the organic ligands (CeO_2_/CuOx@mC). The proposed aptasensor exhibited a low LOD (2.0 fg/mL) within a linear concentration range 0.01 pg/mL–10 ng/mL towards tobramycin [121]. Table 2 summarises some more examples of aptasensors (electrochemical) for the detection of antibiotics (in milk).

### 5.3. Molecularly Imprinted Polymer (MIP)-Based Electrochemical Biosensors

**Figure 14:**
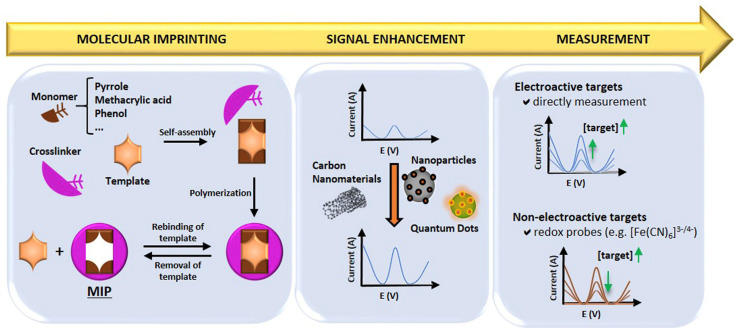
Summary of the preparation procedure, signal enhancement and sensing mechanism for MIPs in MIP-based electrochemical sensors [133]. Reprinted from Ref. [133], Copyright (2021), with permission from Elsevier.

MIPs are synthetic materials with selective binding cavities or recognition sites for a specific target, mimicking natural receptors [131]. MIPs can be synthesised by combining the template molecule and a functional monomer (using inert porogenic solvent), which is polymerised in the presence of an initiator and a crosslinking agent to fix the monomer around the template, generating a 3D polymer network. This is then followed by template removal from the polymeric matrix, thereby leaving specific cavities that complement the template molecule. In this way, molecular memory is generated, and the target analyte/molecule binds with high specificity. Therefore, MIPs acquire the capacity to selectively recognise the target even when other closely related molecules are present, in a similar fashion to the “lock and key” mechanism for enzymes [131,132]. Figure 14 presents a schematic summary of the preparation procedure, signal enhancement strategies, and detection mechanisms employed for MIPs in electrochemical sensors [133].

MIPs have been attracting increasing attention due to the availability of specific receptor sites available for the target analytes. The fabrication processes employ sacrificial spacer techniques that involve the covalent and non-covalent polymerisation of crosslinkers and functional monomers with template molecules [134]. Various nanomaterials, including graphene-based nanomaterials, magnetic nanomaterials, Prussian blue catalytic polymers and nanoparticles, have been employed to improve the sensitivity of MIP-based electrochemical sensors [135,136,137,138,139].

**Figure 15:**
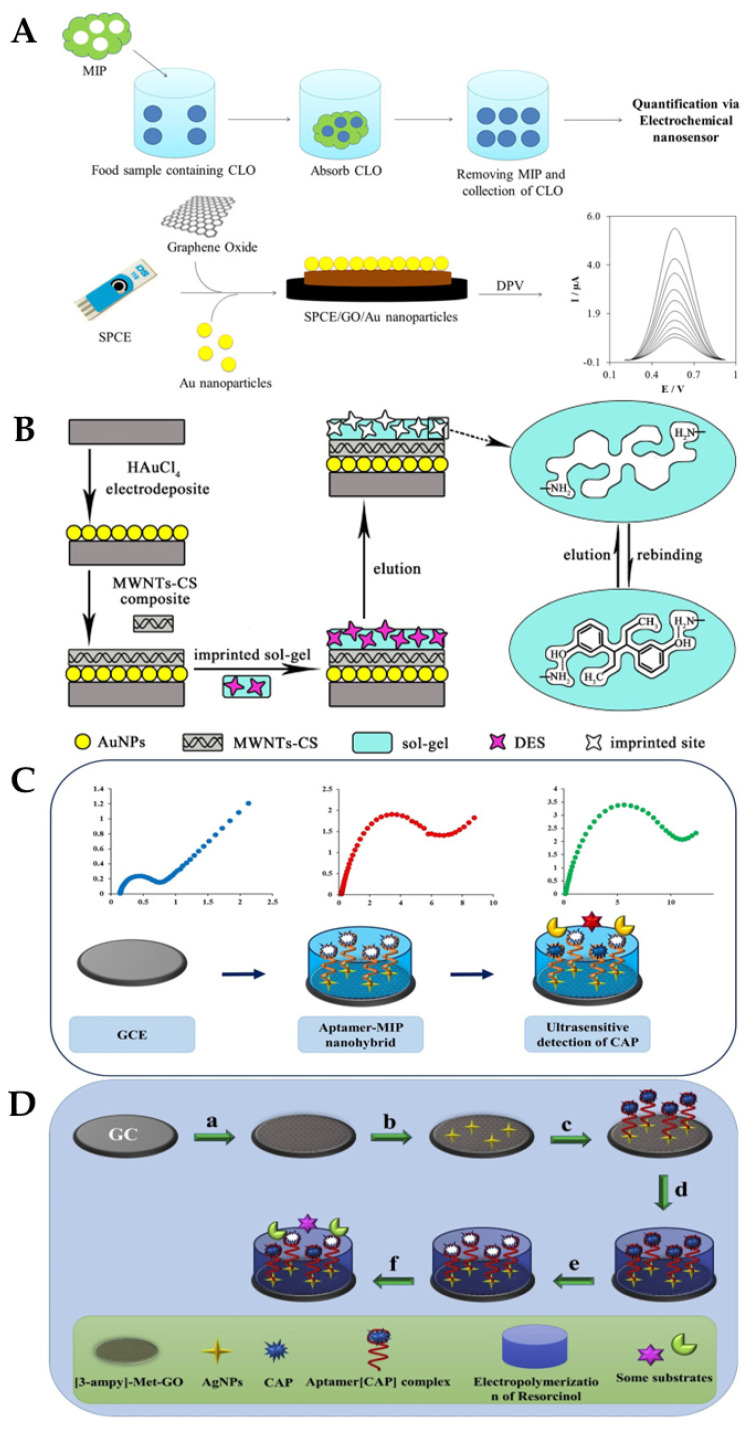
(**A**) Label-free electrochemical detection of the Cloxacillin antibiotic in milk samples based on molecularly imprinted polymer and graphene oxide–gold nanocomposite [140]. Reprinted from Ref. [140], Copyright (2019), with permission from Elsevier. (**B**) Schematic illustration of the fabrication procedure for AuNPs/MWCNTs-CS/sol–gel-MIP/GCE [141]. Reprinted from Ref. [141], Copyright (2017), with permission from Elsevier. (**C**) Schematic diagram of the preparation of the aptamer-MIP nanohybride for CAP detection. Reprinted from Ref. [142], Copyright (2019), with permission from Elsevier. (**D**) Impedimetric ultrasensitive detection of chloramphenicol based on aptamer MIPs using a glassy carbon electrode modified by 3-ampy-RGO and silver nanoparticle [142]: (**a**) covering of 3-ampy-RGO on the GCE surface; (**b**) immobilisation of the AgNPs on the 3-ampy-RGO/GCE; (**c**) covalent attachment of the aptamer[CAP] complex on the AgNP/3-ampy-RGO/GCE surface; (**d**) electropolymerisation of resorcinol on the aptamer[CAP] complex/AgNP/3-ampy-RGO/GCE; (**e**) washing of the modified electrode with washing solution and removal of the CAP; (**f**) addition of CAP as a target and some antibiotic as interferents [142]. Reprinted from Ref. [142], Copyright (2019), with permission from Elsevier.

S. Jafari et al. developed MIP particles using a non-covalent method and assessed the effectiveness of the MIP for the selective removal of cloxacillin (CLO) from biological and aqueous samples, before determining the amount of CLO that had been removed using an electrochemical nanosensor (Figure 15A): a screen-printed electrode enhanced with graphene oxide/AuNPs served as the basis for the electrochemical nanosensor. The best conditions for the elimination of CLO (92%) were reportedly obtained at pH = 8.5, with 89 min as contact time and 0.79 g MIPs. The linear range was from 110 to 750 nM, and a limit of detection of 36 nM was reported [140]. J. Bai et al. described the successful construction of an ultrasensitive electrochemical diethylstilbestrol (DES) sensor (Figure 15B) [141]: on a GCE, gold nanoparticles and a composite made of multi-walled carbon nanotubes and chitosan were incrementally changed (GCE). Under ideal circumstances, an LOD of 24.3 fg/mL and a detection range of 1.0 × 10^−10^–1.0 × 10^−6^ mg/mL were achieved. The recovery rates for the identified DES in milk samples ranged from 91.5% to 106.7% at three concentration levels [141].

M. Roushani et al. developed a biosensor using a dual recognition system based on the molecular imprinting of chloramphenicol (CAP) for selective detection and aptasensing (Figure 15C,D). The CAP complex-amino-aptamer was linked to AgNP/3-ampy-RGO/GCE employing a bonding formation of Ag-N after AgNPs were coated on the 3-ampy-RGO/GCE. Excellent sensing properties were reported as a result of the dual features of the MIPs and aptamers, with a linear range of 1.0 pM–1.0 nM and an LOD of 0.3 pM being achieved in milk samples [142]. Xiaobing Wei and their group proposed a three-dimensional MIP array as an electrochemical sensor for the detection of sulfadimidine (SM2) residues in food. To create the MIP/NiCo_2_O_4_ nanoneedle/3D graphene electrode, polypyrrole (PPy) was electropolymerised in the presence of SM2 and coated onto four nanoneedle arrays. A wide linear range (0.2–1000 ng/mL) and good limit of detection (0.169 ng/mL) were reported [143]. Table 3 summarises some more examples of molecularly imprinted polymer (MIP)-based electrochemical biosensors for the detection of antibiotics in milk.

### 5.4. Enzyme-Based Electrochemical Biosensors

**Figure 16:**
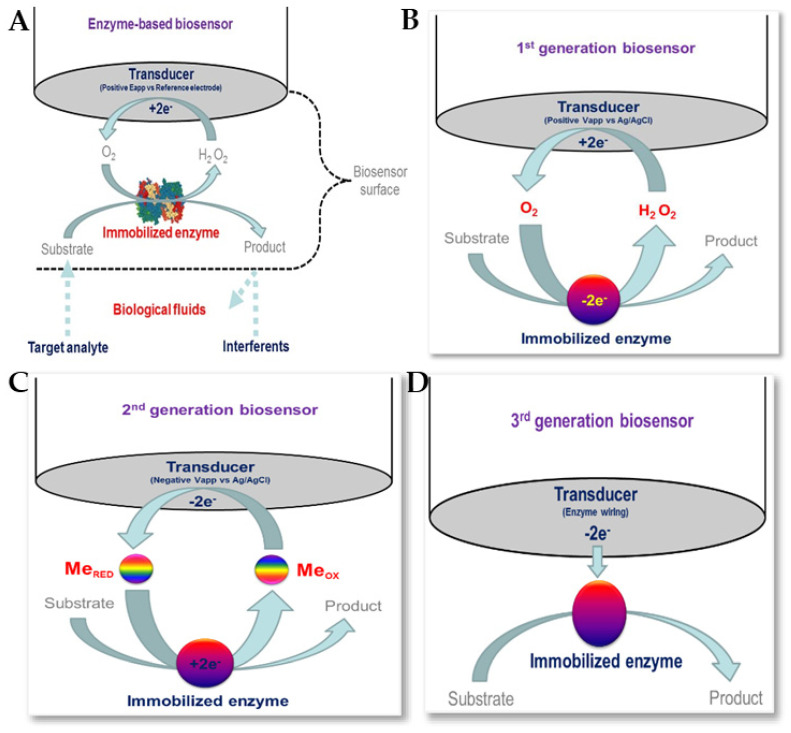
(**A**) Enzyme biosensors for biomedical applications. Schematic representation of (**B**) first-generation, (**C**) second-generation and (**D**) third-generation biosensors [153]. Reproduced under the terms of the CC-BY licence from Ref. [153], Copyright 2016, The Authors, published by MDPI.

Enzymatic biosensors are based on biological recognition, so enzymes must be available to catalyse a specific biochemical reaction, as well as being stable under the required operating conditions (with respect to pH, temperature, etc.) [152,153]. The use of receptors and enzyme labels with nanomaterials has helped in the detection of various antibiotics. Figure 16 shows enzymatic biosensors for biomedical applications, together with schematic representations of first-generation, second-generation, and third-generation biosensors [151]. Several enzymes, such as HRP, PCN, GOx, etc., together with various nanomaterials, including carbon-based nanomaterials and magnetic nanoparticles, have been reported for use in the electrochemical detection of antibiotics [83,154,155].

Penicillinase biosensors for the detection of β-Lactam antibiotics have been reported. β-L antibiotics share a common component in their molecular structures: a four-atom β-Lactam ring. PCN catalyses the opening of the β-Lactam ring, converting penicillin G (PEN) to penicilloic acid and deactivating the molecule’s antibacterial capabilities [156]. Changes in the pH of biosensors for β-L antibiotics have been used to assess the hydrolysis of the β-L ring [93,157,158].

Chen et al. proposed a PEN sensor based on the pH indicator hematein, which was co-immobilised onto a GCE using MWCNTs and PCNase [157]. When PEN was present in a sample, the pH value decreased due to the hydrolysis of PEN to penicilloic acid, which was then catalysed by the PCNase. Hematein (which is pH sensitive) was reduced to hematoxylin once [H]+ was accepted, causing an increase in the electrochemical signal. Interference from the raw milk matrix (through the adsorption of milk proteins and fat onto the electrode surface) was reported. To eliminate this, the fat and proteins were separated from the milk via centrifugation and salting-out, but interference was still evident, and the LOD was 19 ppb.

Wu et al. proposed a PEN biosensor using single-graphene nanosheets (SGCs) [93]. Hematein was attached to the graphene by adsorption, and ionic liquid was added (due to its good biocompatibility) in order to achieve PCNase immobilisation. The activity of the PCNase was reduced due to the accumulation of acidic products at higher PEN concentrations, thereby resulting in decreased sensitivity. A limit of detection of 0.04 pg/L was reported. The limited range of applicability is one of the main drawbacks of pH-dependent biosensors. Ismail and Adeloju proposed a biosensor (potentiometric) for PEN using polytyramine-PCNase non-conducting film on a Pt electrode with a fixed pH (7.0) to ensure optimal enzymatic activity [159]. The biosensor demonstrated a linear range of 3–283 μM, and a minimum detectable concentration of 0.3 μM was achieved for penicillin. This biosensor was utilised for the detection of Amoxycillin, demonstrating an average recovery of 102 ± 6%. Satisfactory penicillin G recoveries were reported in milk samples at concentrations ≥20 ppm. An array based on the change in the oxidation kinetics patterns of lactose (and its metabolites) for the multiplex and rapid detection of common veterinary antibiotics (in raw milk) was reported [160]. Different oxidoreductases were employed to catalyse the lactose oxidation (and its hydrolysis products, galactose and glucose) in different flow channels (sample). The combination of various reaction parameters of various biosensors was used to form a sample pattern for milk, and in the presence of antibiotics, this combination forms a fingerprint for specific antibiotics. An LOD of 50 ppb (PEN) was reported.

**Figure 17:**
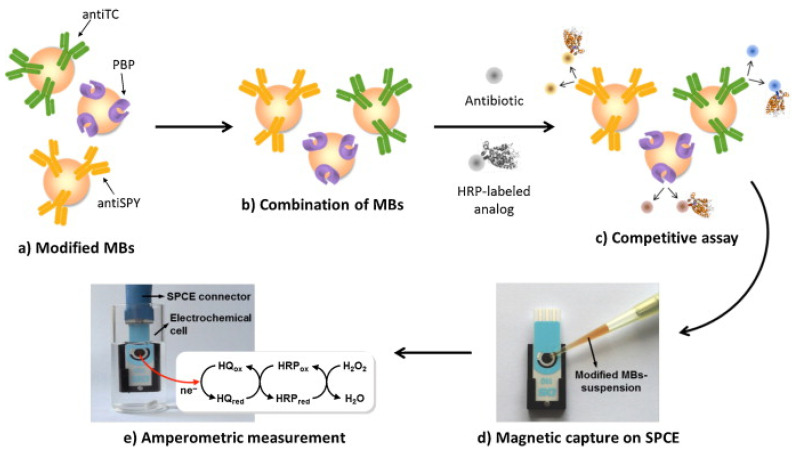
Schematic depiction of the developed multi-antibiotic magnetosensor. The modified MBs (**a**) are commingled together (**b**) and incubated with the sample in the presence of fixed amounts of the three enzyme-labelled analogues, thus establishing a direct competitive assay (**c**); the MBs are then captured on the surface of an SPCE assembled on the magnet holding block (**d**) and the assembly SPCE-magnet holding block immersed in the electrochemical cell is used for the amperometric measurements (**e**) [161]. Reprinted from Ref. [161], Copyright (2014), with permission from Elsevier.

**Figure 18:**
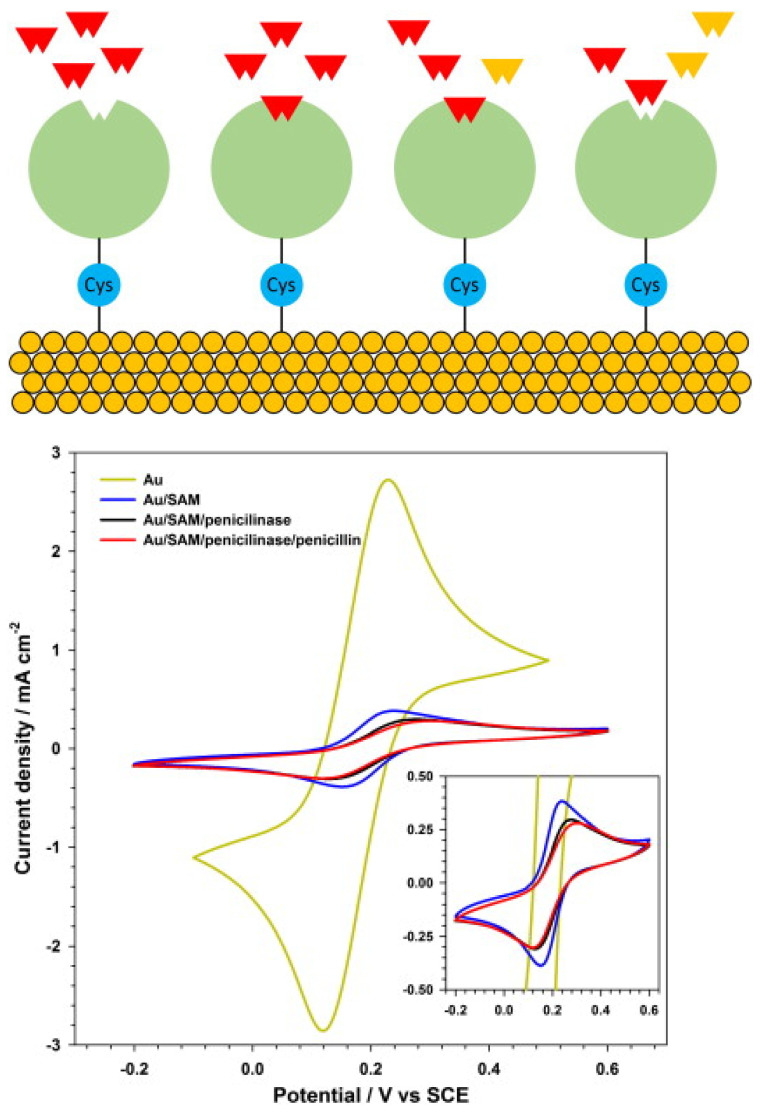
(**Top**) Schematic diagram of the experimental configuration: β-lactamases are connected to the gold electrode surface by cysteine molecules. Penicillin G molecules in solution react with the β-lactamases. (**Bottom**) Cyclic volammetry of a ferrocene solution, 2 mmol L^−1^, in KNO_3_, 1 mol L^−1^, with different electrode surfaces: bare gold; gold and cysteine SAM; gold, cysteine SAM with penicillinase; and gold, cysteine SAM with penicillinase and the analyte penicillin (inset: detail of lower currents) [155]. Reprinted from Ref. [155], Copyright (2014), with permission from Elsevier.

F. Conzuelo et al. reported the monitoring of multiple antibiotic residues (milk) using a screen-printed carbon electrode, MBs (mixture of three target-specifically modified magnetic beads) and direct competitive assays using HRP-labelled tracers (Figure 17). Hydroquinone was applied as a mediator, and H_2_O_2_ as an enzyme substrate, and the method could be used to differentiate between non-contaminated UHT and raw milk samples and samples containing antibiotic residues at their MRLs [161]. L.M. Gonçalves et al. reported a penicillin G biosensor in which penicillinase was immobilised onto a gold electrode via a cysteine self-assembled monolayer (Figure 18). The biosensor was used to amperometrically monitor the catalytic hydrolysis of penicillin, and an LOD of 1.5 µg/L, or 4.5 nM, was reported [155].

### 5.5. Whole-Cell-Based Electrochemical Biosensors (WCBs)

**Figure 19:**
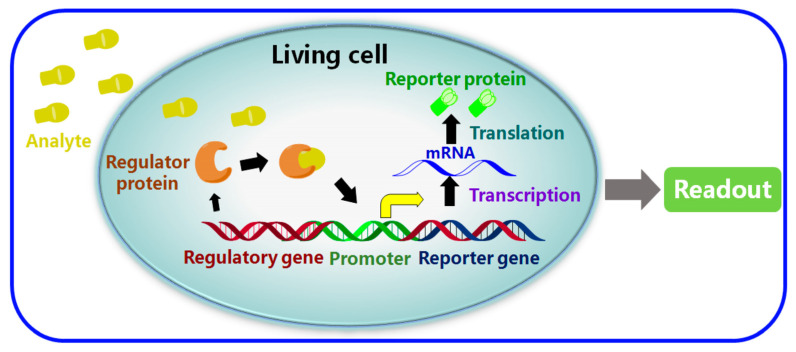
Schematic of a typical whole-cell-based biosensor (WCBs) [162]. Reproduced under the terms of the CC-BY licence from Ref. [162], Copyright 2017, The Authors, published by MDPI.

To create a microbial biosensor for the detection of antibiotics, viable or non-viable cells can be applied with a transducer, where the response of whole cells is used to recognise biologically active agents [47]. Two key parameters related to their performance are the selection of the reporter gene and the sensitivity/selectivity of molecular recognition occurring following the binding of the regulator proteins to the target analytes. As can be seen in Figure 19, which schematically depicts the mechanism of WCBs, following the detection of the target analyte, the next step in the detection process is for the detection to be amplified into an optical/electrical signal using a processor. This can be achieved through the immobilisation and utilisation of bacteria or living whole cells in order to provide molecular recognition [162]. Unicellular microorganisms, especially bacterial cells, are a good candidate for the development of whole-cell-based biosensors [46]. WCBs have been reported for the detection of antibiotics such as tetracyclines [163], beta-lactam antibiotics [164] and quinolones [165]. WCBs possess advantages such as good sensitivity and selectivity, long-term stability, the ability to analyse complex samples without complicated pretreatment, low cost, and in situ detection capability [162]. They can be used to simultaneously detect multiple antibiotics within a timeframe of between 30 min and 3 h. However, when compared to other bioreceptors, WCBs suffer from their narrow/limited detection range. Additionally, unlike chemical reagents or antibodies, living cells are not stable enough, and cannot be stored for a long time without losing viability and responsiveness [46].

Pellegrini et al. proposed an electrochemical microbial biosensor for the detection of tetracyclines (TCs) and quinolones (Qs) based on comparing the measured rate of CO_2_ production to the inhibition of microbial growth (*Escherichia coli*, ATCC 11303) by antibiotics. The evaluation of inhibition after 2 h showed that Qs and TCs were detectable at 25 µg/L. The biosensor was reportedly not sensitive to any of the other antibiotics studied (β-Ls, macrolides, sulfonamides and aminoglycosides) [166]. Ferrini et al. presented a hybrid biosensor in which the microbiological screening of antibacterials was combined with electrochemical detection using *Bacillus stearothermophilus* var. *calidolactis*. Growth was monitored electrochemically by measuring the amount of CO_2_ produced. The presence of antibiotics (β-lactam residues) in milk prevents the growth of the test strain, thereby decreasing the CO_2_ production rate. Variations in CO_2_ production were recorded for 120 min and compared to those in a control milk sample. The limits of detection were found to be at MRL levels [167].

**Figure 20:**
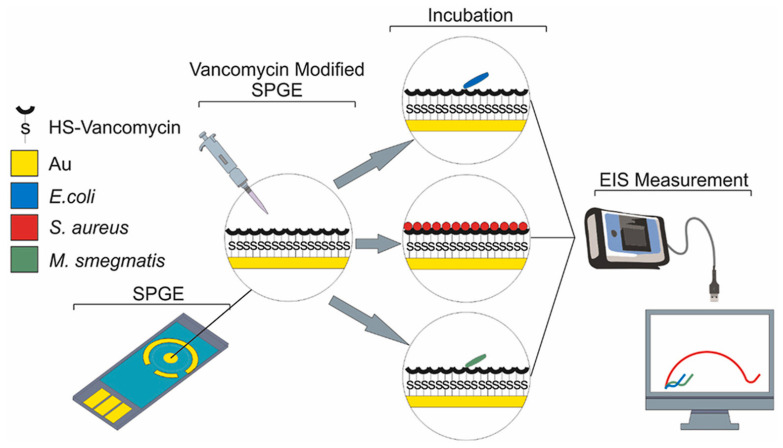
Electrochemical-based “antibiotsensor” for the whole-cell detection of the vancomycin-susceptible bacteria [168]. Reprinted from Ref. [168], Copyright (2021), with permission from Elsevier.

An antibiotic-based biosensor—an ‘Antibiotsensor’—for the specific detection of Gram-positive bacteria using vancomycin-modified screen-printed gold electrodes (SPGEs) was reported by A. Norouz Dizaji et al. (Figure 20) [168]. Electrochemical impedance spectroscopy (EIS) showed a considerable change in impedance upon the binding of HS-Van molecules onto the surface of the electrode. Susceptibility testing was performed using *E. coli*, *Staphylococcus aureus* (*S. aureus*) and *Mycobacterium smegmatis* (*M. smegmatis*) in order to demonstrate its specificity. The change in charge transfer resistance (R_ct_) indicated that vancomycin-susceptible *S. aureus* was successfully and strongly attached onto the SPGE-Van surface, while *M. smegmatis* and *E. coli* did not exhibit any significant attachment behaviour. Different concentrations (10^8^–10 CFU/mL) of *S. aureus* were tested to investigate the sensor’s sensitivity, and a limit of detection of 10^1.58^ CFU/mL and a limit of quantitation of 10^4.81^ CFU/mL were achieved.

### 5.6. Direct Electrochemistry-Based Biosensors

Amoxicillin is known to produce a moderate voltammetric signal, and CNM-modified electrodes can improve this signal. The electrochemical response exhibits a peak due to the oxidation of the phenolic (–OH) to the respective carbonyl group (=O) [74]. In the investigation by Rezaei and Damiri, amoxicillin produced a peak (oxidation) on the MWCNT-modified electrode at approximately 1.0 V and 0.6 V at pH values of 3.0 and 7.5, respectively, and the oxidation current was also much higher compared to that of the bare electrode [169]. Neeraj Kumar et al. reported AuNP-PdNP-ErGO-modified GCE for the detection of amoxicillin [170]. The synergism between Pd and Au nanoparticles on rGO increased the sensitivity and sensor performance by enhancing the charge transfer and the number of adsorption sites on the modified surface of GCE. The peak current of amoxicillin increased with increasing concentration. In addition, the sensor was tested for simultaneous as well as individual analysis of amoxicillin and lomefloxacin, and LOD values of 81 nM and 9 μM were reported for lomefloxacin and amoxicillin, respectively.

Nitrofurans have 5-nitrofuran as their basic structure, and the electroactivity of furan is due to the (irreversible) reduction of the nitro to the nitroso intermediate, which then rapidly reduces to a hydroxylamine group. A simple, low-cost electrochemical sensor for nitrofurantoin (NFT) detection using composite NiFe/*f*-MWCNT was reported by Hwa and Sharma [171]. NFT is an antibiotic used extensively in pharmaceuticals and animal food production. NiFe/*f*-MWCNT composite was synthesised via a hydrothermal mechanism/ultrasonication for catalytic evaluation and NFT detection. The electrocatalyst possesses a high surface area and electron transport, thereby reducing the charge-transfer resistance. The NiFe/*f*-MWCNT screen-printed carbon paste electrode reported an LOD of 0.03 µM for NFT [171]. The NiFe_2_O_4_/rGO nanocomposites were prepared using a hydrothermal method, while the high surface area and electrical conductivity of graphene were reported to improve the signal. The graphene carrier was able to prevent aggregation. The sensor showed a linear correlation with furazolidone concentration in the range 0.1–10.0 μM and 10.0–150.0 μM, and an LOD of 0.05 μM was achieved [172].

## 6. Challenges and Future Perspectives

Biosensors are promising alternatives to existing milk testing procedures. However, there are still limits to the currently available sensors/platforms that require attention, and which should be prioritised in future study. One possibility would be the development of multi-channel microfluidic and multi-target systems for detecting antibiotics/residues in milk before it reaches the bulk tank. Real-world problems include electrode surface biofouling, which may interfere with immobilised molecules and perturb or disrupt the biorecognition process, resulting in erroneous positive/negative readings. With cutting-edge technologies, simultaneous determination possibilities, cost-effective and portable device designs, as well as a good understanding of the proper use of antibiotics, milk analysis will assist in avoiding waste and reducing negligence, thereby enhancing the overall quality. Reliability, robustness and automation are the key features enabling biosensors to become an efficient tool for on-site analysis. Some key challenges for future research are as follows:

Antibiotics analysis: Most studies have focused on the detection of commonly used antibiotics (β-lactams, sulphonamides, tetracyclines and aminoglycosides). Limited studies have attempted biosensing on others, such as colistin, trimethoprim, etc. Antibiotic-spike milk samples have been used in many studies, while analyses of real milk from animals undergoing antibiotic treatment are rare. Real milk samples may contain different antibiotics/residues, as well as other molecules (herbicides, hormones, etc.), which should be considered in real applications of biosensor technologies.

Specificity: To avoid unacceptable amounts of incorrect findings, good biosensor specificity is necessary. The appearance of different chemical structures of the same class of antibiotics, e.g., tetracyclines, could be a serious challenge and contribute to the false results. Prior to immobilisation, highly specialised biological materials must be carefully designed.

Signal amplification and sensitivity: Electrode modification using various nanomaterials has become a widely used technique for signal amplification, and examples were discussed where applicable. Sensitivity could be improved through the use of different sensor components, e.g., nanomaterials, and through various electroanalytical techniques.

Sample handling: Electrochemical sensors offer a rapid speed, and are capable of performing analysis on multiple samples. Microfluidic platforms are an interesting approach, and are capable of offering point-of-care monitoring. Sample volume, simultaneous detection and microfluidic devices are in need of further consideration in the future. Complex biosensing systems require pre-treatment of the milk samples to remove fat/proteins; therefore, on-site analysis seems problematic (as fat/proteins can cause electrode fouling). Sample preparation has not been discussed in detail, likely because most of the proposed electrochemical sensors/biosensors have been developed as proofs-of-concept using spiked samples (sometimes simply by diluting milk with the supporting electrolyte). The validation of parameters such as robustness and the variation of milk matrices is necessary.

Cost: Biosensor cost is an important and ultimate factor from the perspective of both the cost of design and development and recurring consumable costs. Electrochemical devices have advantages in terms of their low production costs, but they are also dependent on market size and scales of economy. Therefore, biosensor approaches that can be adapted to different applications would be more attractive. Future research using 3D-printing technology might lead to the development of low-cost (disposable) high-performance electrochemical sensors and devices for milk sample analysis. It can also provide portable microfluidic devices with embedded electrodes and integrated sample preparation procedures.

Stability and sterilisation: Stability is one of the biggest challenges in biosensor development and application: bio-recognition and the lifetime of the sensors are key factors. In terms of stability, the most promising may be MIP biosensors and aptasensors. There are few data available on the regeneration of biosensor systems. These features would be good to explore in future R&D.

Aptasensors: Studies on the use of a variety of nanomaterials have shown that these materials’ inherent qualities, such as their high pore volumes and surface areas, can improve detectability by increasing their interaction sites. More straightforward production methods are increasing in popularity, and aptasensors, incorporating a low-cost screen-printed electrochemical device platform, could potentially be used to produce commercial version of the sensors. The simple design of aptasensors could be a benchmark for consideration in future developments for the analysis of complex samples. The reproducibility of fabrication and incubation time (faster analysis) also need to be considered for aptasensors, along with rigorous testing of the sample preparation procedures with appropriate calibration data analysis.

MIPs: The combination of MIPs with magnetic particles can facilitate the removal of antibiotics from samples, which can be easily incorporated with electrochemical detection systems through the following two steps: (i) the extraction of antibiotics/residues from the bulk solution using functionalised MIP particles; and (ii) magnetic transfer of MIP particles to the electrode surface for detection. MIP-based methods have the potential to improve electrode lifetime and stability in real-world applications. The reuse of MIPs is a major challenge, and should be further explored in future research.

Immunosensors: The specificity/selectivity resulting from the antibodies used is an important aspect of immunosensors. Polyclonal antibodies are well known to be less specific than monoclonal antibodies, and so could result in insufficient specificity and significant cross-reactivity towards antibiotics. Monoclonal antibodies are highly specific, but producing them is complicated, demanding, and costly. More studies on the use of nanostructured materials in immunosensor manufacturing and multiple labelling would be beneficial. Sample preparation and matrix, specificity/sensitivity, and analysis time are all important aspects to consider.

Whole-cell biosensors: WCBs can give real-time, fast, and unique data streams describing the cell’s homeostatic condition with high sensitivity/selectivity. Stability, reproducibility, and analysis time, as well as multiple analysis aspects, would be interesting to explore further, such as the fabrication of specific and multifunctional whole-cell biosensors for rapid and real-time detection in real environments.

Enzyme-based biosensors: Enzymatic biosensors are highly selective and can be used for reliable and continuous monitoring. They can detect analytes at low concentrations due to their specificity; however, various enzymes are dependent on substrate concentrations, pH levels and temperatures. Because the presence of inhibitors may affect their catalytic properties and performance, biosensors must be designed with the target analyte and matrix complexity in mind. The most typical problems are fouling agents and interference and inactivation due to denaturation or aggregation caused by changes in pH and temperature. Greater attention is required to be paid to enzyme stability and detection efficacy, and highly selective and stable enzyme-based biosensors would be highly desirable, with expected application in real sample analysis in the future.

## 7. Conclusions

The extensive use of antibiotics in agriculture or in animal feed to improve growth and productivity have resulted in significant contamination of human food items. According to the WHO, “antibiotic resistance” is a severe economic and social danger, threatening public health, and the COVID-19 pandemic was a recent wake-up call, challenging us to improve our understanding and prepare for the future. Currently, chromatography techniques (e.g., chromatographic techniques, LC-MS, HPLC) coupled with various detectors are being used for the analysis of antibiotics. To minimise the problems associated with antibiotics (AMR development) and current analytical methods, electrochemical platforms have been investigated for the detection of antibiotics/residues, and are capable of providing a cost-effective, portable and rapid alternative. Despite the advances in this area, further research has to be performed to improve electrochemical sensors, such as through the use of multifunctional nanomaterials to guarantee effective detection, portability and consistency. This review summarised electrochemical biosensors for the detection of antibiotics in milk/milk products and presented an introduction to antibiotics and AMR followed by electrochemical biosensor approaches based on (i) immunosensors, (ii) aptamers, (iii) MIPs, (iv) enzymes, (v) whole cells, and (vi) direct electrochemistry. The role of nanomaterials or advanced sensor fabrication materials is discussed wherever necessary. Finally, the challenges and future perspectives were discussed.

The simultaneous antibiotics monitoring of milk samples should be considered in future investigations, with sensitivity and selectivity appropriate to regulatory requirements. When it comes to portable platforms for simultaneous antibiotic detection, advances in the design of multi-analyte sensors require careful attention. Electrochemical sensors/biosensors hold great promise because they can be constructed on miniaturised multiplexed sensing platforms. The utilisation of innovative materials/biomaterials and functional nanomaterials has the potential to improve sensitivity, selectivity, biocompatibility, and overall performance, thereby opening new avenues for future the analysis of antibiotics.

## Figures and Tables

**Table 2:** Electrochemical aptasensors for the detection/determination of antibiotics (in milk).

Antibiotics	Biorecognition Component	Working Electrode	Detection Method/Mode	Linear/Dynamic Range	LOD	Sample Type	Ref.
Tetracycline	Aptamer	Aptamer/GNP/MNP/PGE	EIS	1 pM to 1 µM	0.03 pM	Milk of cow, sheep, goat, and buffalo	[118]
	5′-SH-CCC CCG GCA GGC CAC GGC TTG GGT TGG TCC CAC TGC GCG-3′						
Oxytetracyclines	5′-TCA CGT TGA CGC TGG TGC CCG GTT GTG GTG GGA GTG TTG TGT- (CH_2_)_6_- NH_2_-3′	GCE, capture beads (anti-ssDNA Ab/Dynabeads) & Apts-MNM	SWV	0.5 to 5 × 10^4^ pM	0.18 pM	Milk	[114]
Kanamycin	5′-TCT GGG GGT TGA GGC TAA GCC GAC- (CH_2_)_6_- NH_2_-3′	GCE, capture beads (anti-ssDNA Ab/Dynabeads) & Apts-MNM	SWV	0.5 to 5 × 10^4^ pM	0.15 pM	Milk	[114]
Tobramycin	5′-Bio-GGCACGAGG UUUAGCUACACUCGUGCC-NH_2_-3′	AuNSs/Gold electrode	DPV	1 to 10^4^ nM	0.49 nM	Spiked milk	[119]
Streptomycin	5′-TAG GGA ATT CGT CGA CGG ATC CGG GGT CTG GTG TTC TGC TTT GTT CTG TCG GGT CGT CTG CAG GTC GAC GCA TGC GCC G-thiol-3′	MWCNTs–CuO–AuNPs/PCNRs/GCE	DPV	0.05 to 300 ppb	0.036 ppb	Milk & Honey	[122]
Penicillin	5′-thiol-(CH_2_)_6_-CTG AAT TGG ATC TCT CTT CTT GAG CGA TCT CCA CA-3′	pDNA/AuNPs/ECNF mat electrode	CV	1 to 400 ppb	0.6 ppb	Spiked fat-free milk	[123]
Aminoglycosides	5′-CGGATCCCCAGCT-CGGGGTGCTATGGAGG-CTGTATCGGAGACCTGCAGG-3′	Ti_3_C_2_ MXene/OMC-CS/SPCE	DPV	10 to 2 × 10^3^ nM	3.51 nM	Spiked milk	[120]
Ciprofloxacin	5′ –ATACCAGCTTATTCAA-TTGCAGGGTATCTG-AGGCTTGATCTACT-AAATGTCGTGGGGCA-TTGCTATTGGCGTTGA-TACGTACAATCGTAA TCAGTTAG-3′	Apt/3D Au-PAMAM/rGO/GCE	DPV/SWV	1 nM to 1 µM	1 nM (LLOQ)	spiked milk	[124]
Penicillin-G	5′GGGTCTGAGGAGTG-CGCGGTGCCAGTGAGT-3′	Gold functionalised electrode	SWV	5 nM to 5 µM	1.7 nM	Spiked milk	[125]
Kanamycin	3′-NH2-TGG GGG TTG AGG CTA AGC CGA-C-5’	GCE covered with carbon black and Calix arene-bearing lactic fragments, aminated aptamer covalently attached via carbodiimide binding	EIS	0.7–50 nM	0.3 nM	Spiked milk	[126]
Oxytetracycline	5′-NH2-GGA ATT CGC TAG CAC GTT GAC GCT GGT GCC CGG TTG TGG TGC GAG TGT TGT GTG GAT CCG AGC TCC ACG TG-3′	GCE grafted with diazonium salt, followed by aptamer attachment by carbodiimide binding	DPV	10^−3^ to 100 ppm	0.229 ppb	Spiked milk	[127]
Tobramycin	5′-ACUUGGUUUAGGUAAUGAGU-3′	CeO_2_/CuO_x_@mC nanocomposite	EIS	0.01 to 10^4^ ppt	2.0× 10^−3^ ppt	Spiked milk/human serum	[121]
Ampicillin	3′-thiol-modified 40 nucleotides (ATW0001-GO3-GN3-100) with a 10 bases 3′ spacer and without any 5′ modification	Inkjet-printed AgNPs	EIS	10^2^ to 10^4^ ppm	10 ppm	Milk	[128]
Kanamycin & Streptomycin	5′-NH_2_-ACGACCCGACAGAACAAAGCAGAACA CCAGACCCCAAAAAAAAAATCGGCTTAGCCTCAACCCCCATCT-3′	Multiplexed graphitised multi-walled carbon nanotubes/carbon nanofibers-gold nanoparticles aptasensor (MWCNTGr/CNFs-AuNPs/SPCE)	DPV	10^2^ to 10^5^ pM	74.50 pM (KAN) & 36.45 pM (STR)	Diluted milk	[129]
17β-estradiol (E2)	5′-Thiol-TTTTTTTTTTTTTTTGCTTCCAGCTTATTGAATTACACGCAGAGGGTA-3′ (split1) and 5′- GCGGCTCTGCGCATTCAATTGCTGCGCGCTGAAGCGCGGAAGCTTTTTTTTTTTT-Thiol-3′ (split2)	Screen-printed gold electrode	DPV	3 to 300 pM 300-9000 pM	0.7 pM	Diluted spiked milk samples	[130]

**Table 3:** Molecularly imprinted polymer (MIP)-based electrochemical biosensors for the detection of antibiotics in milk.

Analyte	Sensing Scheme	Detection Method	Linear Range	LOD	Sample	Ref.
Ciprofloxacin	Ch-AuMIP/GCE	DPV	1 to 100 µM	0.21 µM	Mineral & tap water, milk and pharmaceuticals	[144]
Cloxacillin	MIP-GO-AuNPs/SPE	DPV	0.11 to 0.75 µM	0.036 µM	PBS/milk	[140]
Kanamycin	MMIP/CE (MIP-MWCNTs-Fe_3_O_4_/CE)	DPV	10^−4^ to 1 µM	2.3 × 10^−5^ µM	PBS/milk/liver	[145]
Sulfanilamide	MIP/GO/GCE	SWV	10 to 1000 ppb	-	Buffer	[146]
Streptomycin	MIP/Gold electrode	DPV	0.01 to 10 ppb	0.007 ppb	PBS/milk/honey	[147]
Neomycin	MIPs/GR-MWCNTs/CS-SNP/gold electrode.	Amperometry	0.009 to 7 µM	7.63 × 10^−3^ µM	Standard solution/milk/honey	[148]
Chloramphenicol	aptamer-MIP/AgNP/3-ampy-RGO/GCE	EIS	1 × 10^−6^ to 1 × 10^−3^ µM	0.3 × 10^−6^ µM	PBS/milk	[142]
17β-estradiol (Steroid)	MIP/NPGL/Au	CV	1 × 10^−6^ to 10 µM	1 × 10^−7^ µM	Food samples	[149]
Sulfamethoxasole	PDA-MIP/gold electrode	Amperometry	0.8 to 170 µM	0.8 µM	PBS/milk	[150]
Sulfadimidine	MIP-NiCo_2_O_4_/3D graphene sensor	DPV	0.2 to 1000 ppb	0.169 ppb	Spiked milk samples	[143]
Tetracycline	BMMIP/GCE	DPV	0.025 to 500 ppm	0.025 ppm	Buffer/milk	[151]

## Data Availability

Not applicable.

## Abbreviations

WHO:	World Health Organization
EMA:	European Medicines Agency
AMR:	Antimicrobial resistance
CIAs:	Critically important antibiotics
MRL:	Maximum residue limit
LOD:	Limit of detection
RSD:	Relative standard deviation
SPR:	Surface-plasmon resonance
PEN:	Penicillin
PCN:	Penicillinase
PENG:	Penicillin G
PBP:	Penicillin-binding protein
CAP:	Chloramphenicol
CLO/CLOX:	Cloxacillin
TOB:	Tobramycin
KAN:	Kanamycin
AMP/AMPI:	Ampicillin
AMOX:	Amoxicillin
CEF:	Cefapirin
OXA:	Oxacillin
TCs:	Tetracyclines
Qs:	Quinolones
DES:	Diethylstilbestrol
HRP:	Horseradish peroxidase
HRP-AMP:	Horseradish peroxidase-labelled ampicillin
GOx:	Glucose oxidase
GCE:	Glassy carbon electrode
SPE:	Screen-printed electrode
SPCEs:	Screen-printed carbon electrodes
SPGEs:	Screen-printed gold electrodes
m-GEC:	Magnetic graphite–epoxy composite
WE:	Working electrode
RE:	Reference electrode
CV:	Cyclic voltammetry
DPV:	Differential pulse voltammetry
SWV:	Square-wave voltammetry
EIS:	Electrochemical impedance spectroscopy
MIP:	Molecularly imprinted polymer
rGO:	Reduced graphene oxide
NFT:	Nitrofurantoin
AuNPs:	Gold nanoparticles
MWCNTs:	Multi-walled carbon nanotubes
SGCs:	Single-graphene nanosheets
MNP:	Magnetic nanoparticles
MoS_2_:	Molybdenum disulfide
MOF:	Metal–organic framework
SAM:	Self-assembled monolayer
WCBs:	Whole cell-based biosensor
HPLC:	High performance liquid chromatography
LC-MS:	Liquid chromatography-mass spectroscopy
GC:	Gas chromatography-mass spectroscopy
HQ:	Hydroquinone
DFR:	Dry film photoresist
QDs:	Quantum dots
MBs:	Magnetic beads
MB:	Methylene blue
ELAs:	Enzyme-linked assays
ELISA:	Enzyme-linked immunosorbent assay
EDC:	1-Ethyl-3-(3-dimethylaminopropyl)carbodiimide
PPy:	Polypyrrole
PAMAM:	Poly(amidoamine)
SM2:	Sulfadimidine
ppm:	Parts per million
ppb:	Parts per billion
ppt:	Parts per trillion
R_ct_:	Charge transfer resistance
